# Supplementary material for: Characterization of Ginsenosides from the Root of Panax ginseng by Integrating Untargeted Metabolites Using UPLC-Triple TOF-MS
Source: Molecules. 2023 Feb 22;28(5):2068. doi: 10.3390/molecules28052068 (PMC10004652; doi:10.3390/molecules28052068)
Supplement: Supplementary file 1 [file molecules-28-02068-s001.zip › molecules-2197177-supplementary.pdf]

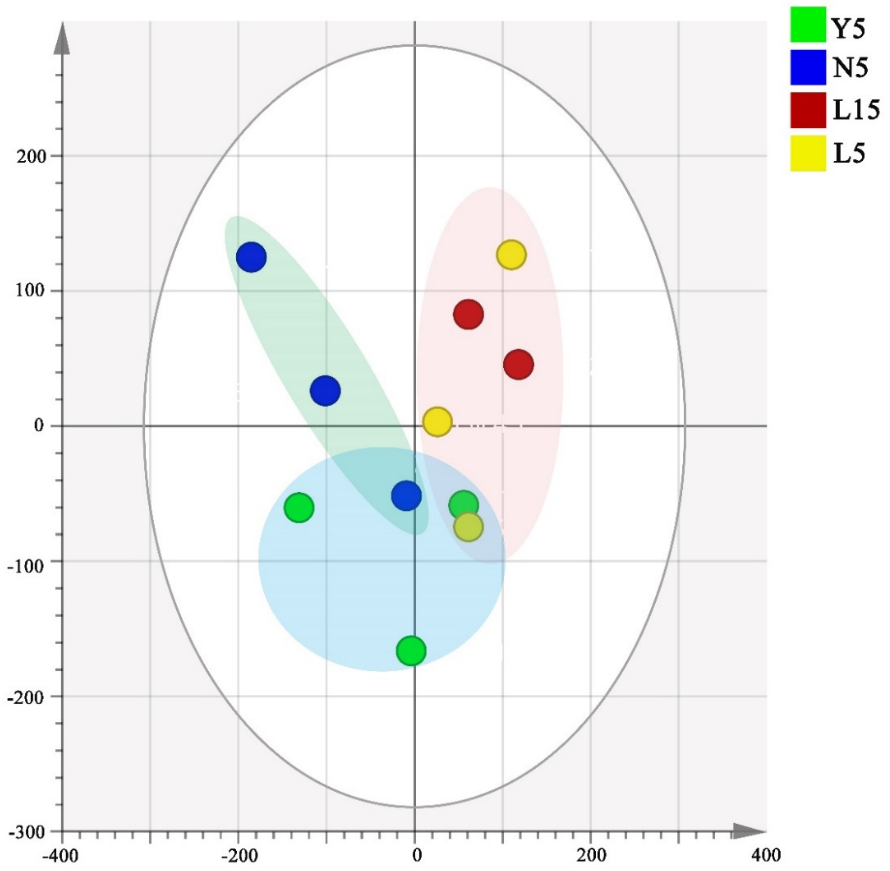

Figure S1: The PCA analysis for the four groups of samples.

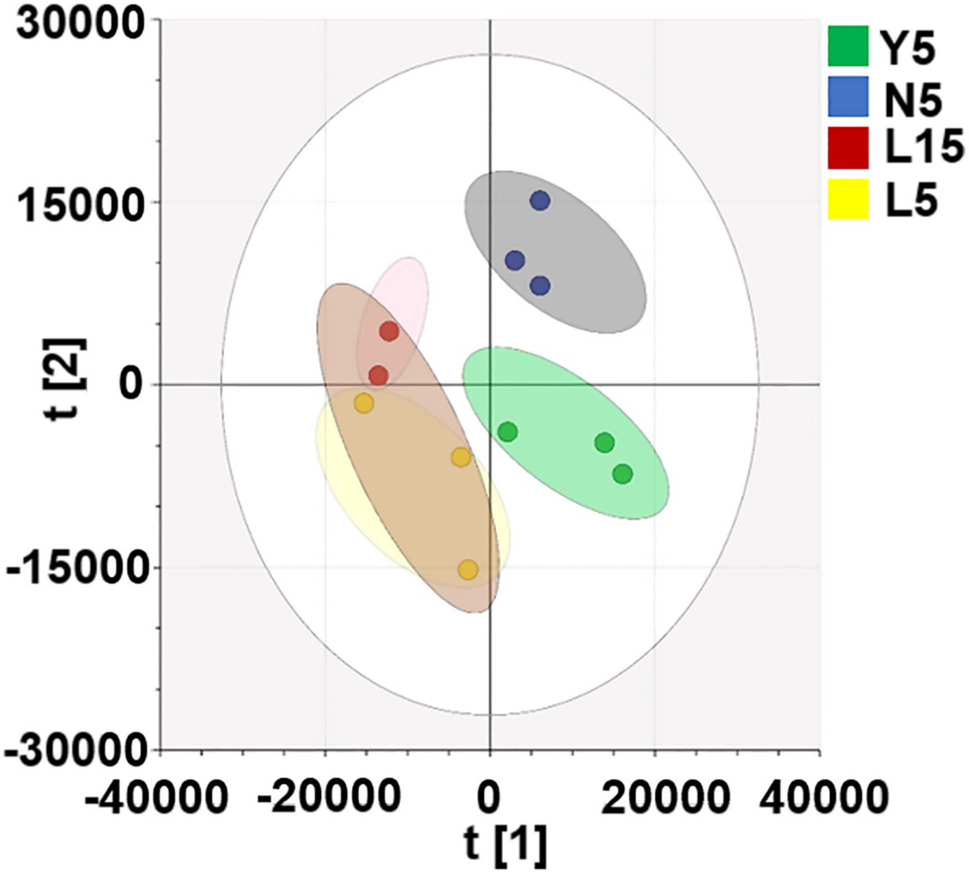

Figure S2: The OPLS-DA analysis for the four groups of samples.

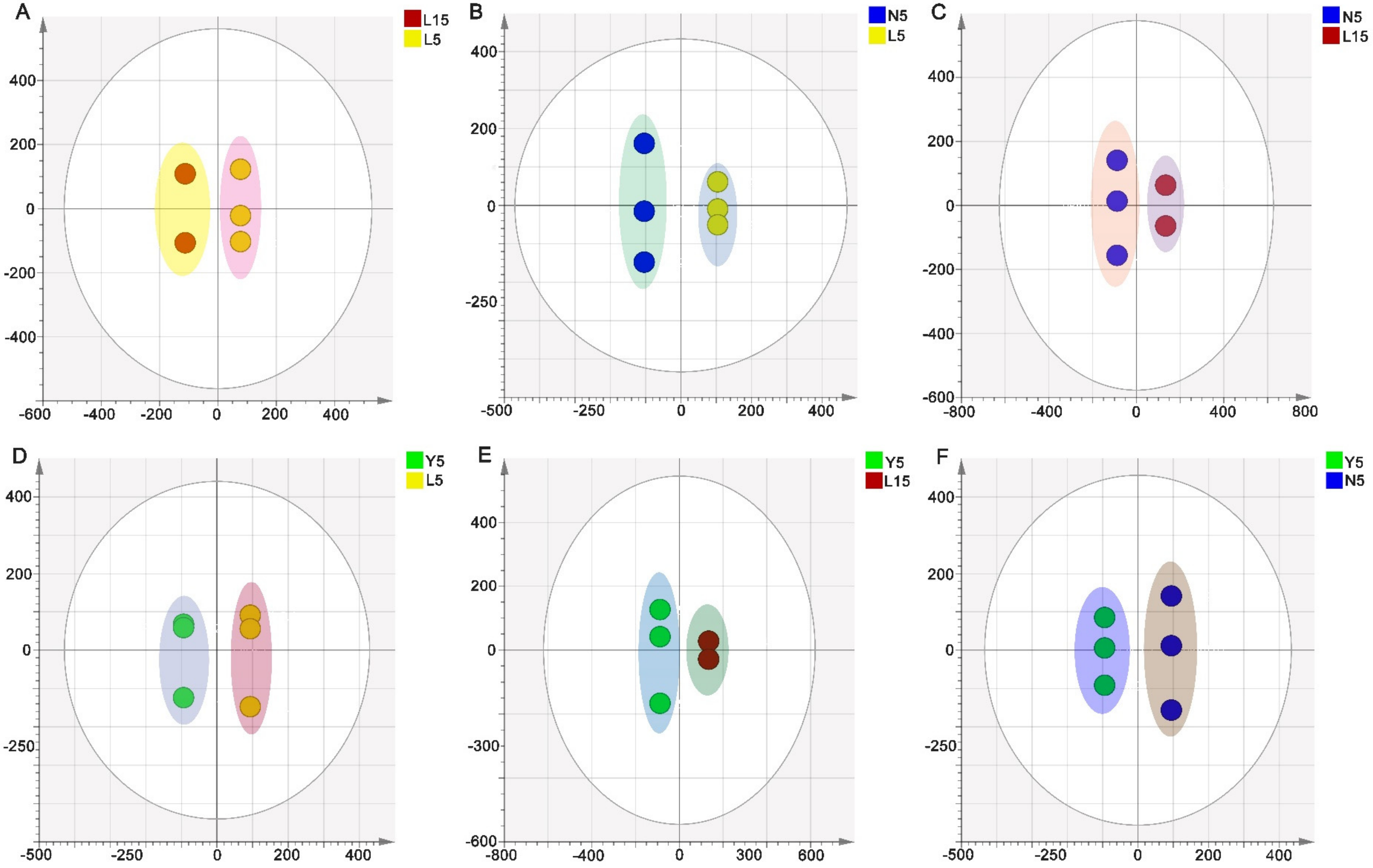

Figure S3: The OPLS-DA analysis for the pairwise comparison of the four groups of samples.

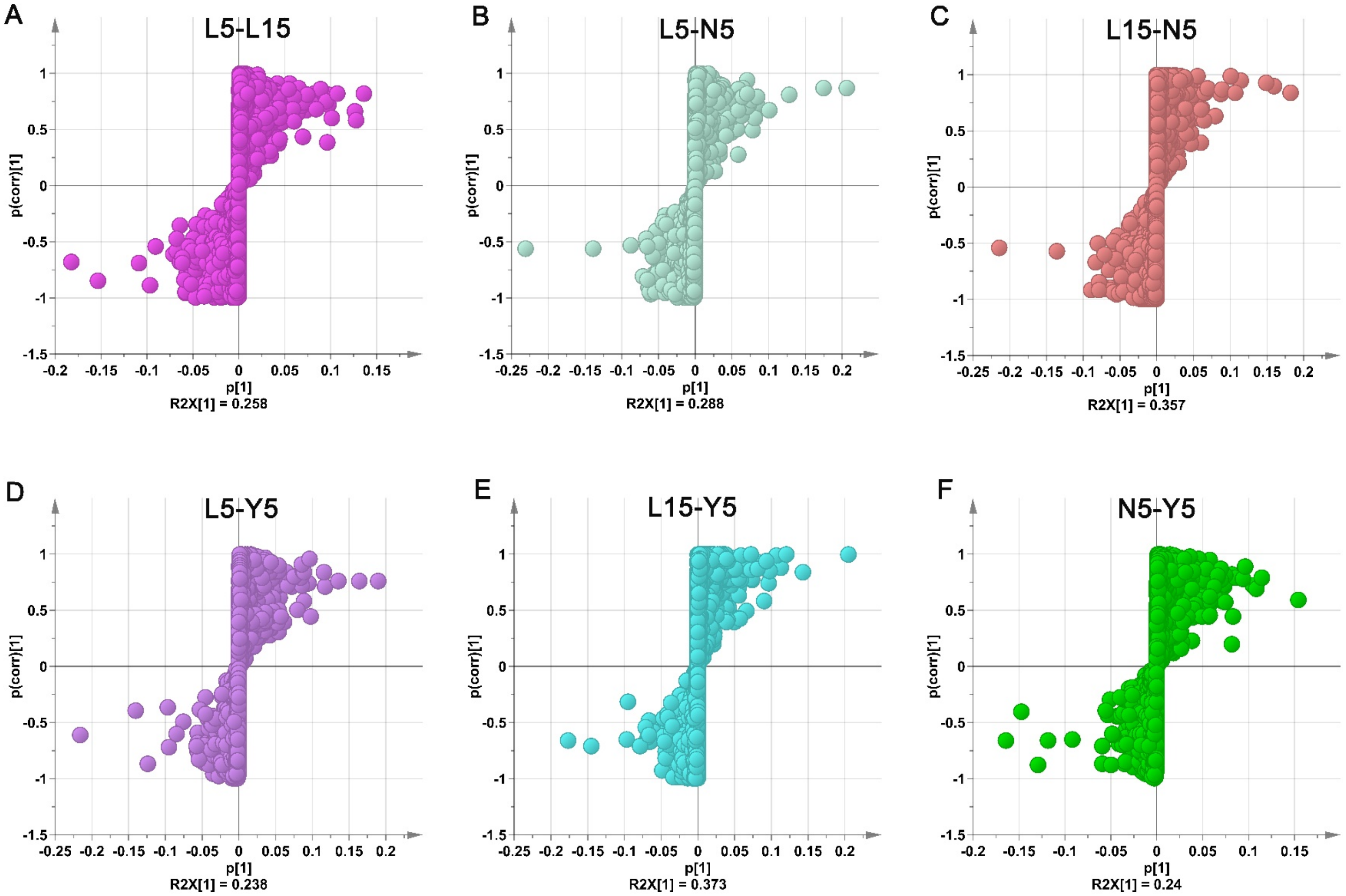

Figure S4: The S-Plot analysis for the pairwise comparison of the four groups of sample.

Table S1 MS and MS/MS of the 237 known ginsenosides

Retention Time	Found at Mass	MS/MS	Name	Formula	Theoretical Mass (Da)	Sources	Error (ppm)
1.18	861.4858	861.4879[M+FA-H]⁻ 815.4800[M-H]⁻ 653.4251[M-H-Glc]⁻ 635.4230[M-H-Glc-H_2O]⁻ 491.3715[M-H-2Glc]⁻	Panajaponol A/isomer	$C_{42}H_{72}O_{15}$	816.4871	L5、L15、Y5、N5	-0.86
1.25	863.5005	863.5007[M+FA-H]⁻ 817.4953[M-H]⁻ 655.4458[M-H-Glc]⁻ 493.3921[M-H-2Glc]⁻	20-S-Rf_3/ isomer	$C_{42}H_{74}O_{15}$	818.5028	L5、L15、Y5、N5	-0.37
1.37	1003.5493	1003.5530[M-H]⁻ 961.5460[M-H-Ac]⁻ 943.5340[M-H-Ac-H_2O]⁻ 799.4966[M-H-Ac-Glc]⁻ 637.4406[M-H-Ac-Glc-Glc]⁻ 475.3464[M-H-Ac-Glc-Glc-Glc]⁻	6-acetyl ginsenoside-Rg_3/isomer	$C_{50}H_{84}O_{20}$	1004.5556	L5、L15、Y5、N5	-1.49
1.62	841.4954	841.4990[M-H]⁻ 799.4915[M-H-Ac]⁻ 781.4738[M-H-Ac-H_2O]⁻ 679.4414[M-H-Glc]⁻ 637.4319[M-H-Ac-Glc]⁻ 619.4212[M-H-Ac-Glc-H2O]⁻ 475.3776[M-H-Ac-Glc-Glc]⁻	noto-Rt/isomer	$C_{44}H_{74}O_{15}$	842.5028	L5、L15、Y5、N5	-0.48
1.62	827.4795	827.4811[M+FA-H]⁻ 781.4744[M-H]⁻ 619.4250[M-H-Glc]⁻	G-Rh_{15}/isomer	$C_{42}H_{70}O_{13}$	782.4816	L5、L15、Y5、N5	-0.77
1.63	987.5533	987.5576[M-H]⁻ 945.5478[M-H-Ac]⁻ 927.5359[M-H-Ac-H_2O]⁻ 799.4704[M-H-Ac-Rha]⁻ 637.4245[M-H-Ac-Rha-Glc]⁻ 475.3881[M-H-Ac-Rha-Glc-Glc]⁻	Pseudoginsenoside Rs_1/isomer	$C_{50}H_{84}O_{19}$	988.5607	L5、L15、Y5、N5	-0.41

1.79	1031.544	1031.5449[M-H]⁻ 987.5586[M-H-CO_2]⁻ 945.5471[M-H-CO_2-Ac]⁻ 927.5362[M-H-CO_2-Ac-H_2O]⁻ 799.4916[M-H-CO_2-Ac-Rha]⁻ 637.4381[M-H-CO_2-Ac-Rha-Glc]⁻ 475.3786[M-H-CO_2-Ac-Rha-1Glc]⁻	mRd isomer	$C_{51}H_{84}O_{21}$	1032.5505	L5、L15、Y5、N5	-1.26
1.87	861.4859	861.4826[M+FA-H]⁻ 815.4788[M-H]⁻ 653.4314[M-H-Glc]⁻ 491.3729[M-H-2Glc]⁻	Panajaponol A/isomer	$C_{42}H_{72}O_{15}$	816.4871	L5、L15、Y5	0.61
1.89	885.4879	885.4878[M-H]⁻ 841.4982[M-H-CO_2]⁻ 799.4874[M-H-CO_2-Ac]⁻ 781.4768[M-H-CO_2-Ac-H_2O]⁻ 679.4427[M-H-CO_2-Glc]⁻ 637.4336[M-H-CO_2-Ac-Glc]⁻ 619.4224[M-H-CO_2-Ac-Glc-H_2O]⁻ 475.3784[M-H-CO_2-Ac-2Glc]⁻	mRg_1 isomer	$C_{45}H_{74}O_{17}$	886.4926	L5、L15、Y5、N5	-3.50
1.93	1123.5858	1123.5959[M-H]⁻ 961.5497[M-H-Glc]⁻ 799.4793[M-H-2Glc]⁻ 637.4076[M-H-3Glc]⁻ 475.3864[M-H-4Glc]⁻	Notoginsenoside Fh_7 isomer	$C_{54}H_{92}O_{24}$	1124.5979	L5、L15	3.83
1.95	799.4848	799.4875[M-H]⁻ 637.4325[M-H-Glc]⁻ 475.3799[M-H-2Glc]⁻	Rg_1 isomer	$C_{42}H_{72}O_{14}$	800.4922	L5、L15、Y5、N5	-0.50
1.96	945.5399	945.5496[M-H]⁻ 799.4873[M-H-Rha]⁻ 637.4582[M-H-Glc]⁻ 475.3807[M-H-Rha-Glc-Glc]⁻	G-Re isomer	$C_{48}H_{82}O_{18}$	946.5501	L5、L15、Y5、N5	2.54
2.21	885.4873	885.4875[M-H]⁻ 841.4975[M-H-CO_2]⁻ 799.4869[M-H-CO_2-Ac]⁻ 637.4317[M-H-CO_2-Ac-Glc]⁻ 475.3839[M-H-CO_2-Ac-2Glc]⁻	mRg_1 isomer	$C_{45}H_{74}O_{17}$	886.4926	L5、L15、Y5、N5	-2.82
2.21	841.498	841.4979[M-H]⁻ 799.4859[M-H-Ac]⁻ 637.4293[M-H-Ac-Glc]⁻ 475.3849[M-H-Ac-2Glc]⁻	noto-Rt/isomer	$C_{44}H_{74}O_{15}$	842.5028	L5、L15、Y5、N5	-3.57
2.25	987.5548	987.5547[M-H]⁻ 945.5413[M-H-Ac]⁻ 927.5322[M-H-Ac-H_2O]⁻ 799.4837[M-H-Ac-Rha]⁻ 783.4981[M-H-Ac-Glc]⁻ 781.4879[M-H-Ac-Rha-H_2O]⁻ 637.4303[M-H-Ac-Glc-Rha]⁻ 475.3716[M-H-Ac-Glc-Rha-Glc]⁻	Pseudoginsenoside Rs_1/isomer	$C_{50}H_{84}O_{19}$	988.5607	L5、L15、Y5、N5	-1.92

2.34	827.4815	827.4824[M+FA-H]⁻ 781.4755[M-H]⁻ 619.4175[M-H-Glc]⁻ 457.3386[M-H-2Glc]⁻	G-Rh_{15}/isomer	$C_{42}H_{70}O_{13}$	782.4816	L5、L15、Y5、N5	-2.18
2.72	799.4849	799.4803[M-H]⁻ 637.4140[M-H-Glc]⁻ 475.3608[M-H-Glc-Glc]⁻	Rg_1 isomer	$C_{42}H_{72}O_{14}$	800.4922	L5、L15、Y5、N5	-0.63
2.74	885.4877	885.4880[M-H]⁻ 841.4993[M-H-CO_2]⁻ 799.4819[M-H-CO_2-Ac]⁻ 637.4325[M-H-CO_2-Ac-Glc]⁻ 619.4209[M-H-CO_2-Ac-Glc-H_2O]⁻ 475.3920[M-H-CO_2-Ac-2Glc]⁻	mRg_1 isomer	$C_{45}H_{74}O_{17}$	886.4926	L5、L15、Y5、N5	-3.28
2.77	841.4953	841.5016[M-H]⁻ 799.4834[M-H-Ac]⁻ 781.4688[M-H-Ac-H_2O]⁻ 637.4381[M-H-Ac-Glc]⁻ 619.4221[M-H-Ac-Glc-H_2O]⁻ 475.3735[M-H-Ac-2Glc]⁻	noto-Rt/isomer	$C_{44}H_{74}O_{15}$	842.5028	L5、L15、Y5、N5	-0.36
2.82	863.4998	863.4849[M+FA-H]⁻ 817.4966[M-H]⁻ 655.4481[M-H-Glc]⁻ 493.3895[M-H-2Glc]⁻	20-S-Rf_3/ isomer	$C_{42}H_{74}O_{15}$	818.5028	L5、L15、Y5、N5	-1.96
2.83	987.5541	987.5572[M-H]⁻ 945.5459[M-H-Ac] 927.5299[M-H-Ac-H_2O]⁻ 799.4982[M-H-Ac-Rha]⁻ 781.4555[M-H-Ac-Rha-H_2O]⁻ 637.4408[M-H-Ac-Rha-Glc]⁻ 475.3925[M-H-Ac-Rha-Glc]⁻	20-S-Rf_3/ isomer	$C_{50}H_{84}O_{19}$	988.5607	L5、L15、Y5、N5	-1.22
2.87	1031.5437	1031.5450[M-H]⁻ 987.5545[M-H-CO_2]⁻ 927.5376[M-H-CO_2-Ac-H_2O]⁻ 799.4965[M-H-CO_2-Ac-Rha]⁻ 637.4275[M-H-CO_2-Ac-Rha-Glc]⁻ 475.3859[M-H-CO_2-Ac-Rha-2Glc]⁻	mRd isomer	$C_{51}H_{84}O_{21}$	1032.5505	L5、Y5、N5	-0.97
3	861.4824	815.4809[M-H]⁻ 653.4293[M-H-Glc]⁻ 635.4135[M-H-Glc-H_2O]⁻ 491.3729[M-H-2Glc]⁻	Panajaponol A/isomer	$C_{42}H_{72}O_{15}$	816.4871	L5、L15、Y5、N5	-1.96
3.2	961.5397	961.5397[M-H]⁻ 799.4887[M-H-Glc]⁻ 637.4224[M-H-2Glc]⁻ 475.3553[M-H-3Glc]⁻	Re_1 isomer	$C_{48}H_{82}O_{19}$	962.545	L5、L15、Y5、N5	-2.60
3.3	947.52	947.5208[M-H]⁻ 815.4781[M-H-Xyl/Ara]⁻ 653.4309[M-H-Xyl/Ara-Glc]⁻ 491.3935[M-H-Xyl/Ara-2Glc]⁻	Vinaginsenoside R_6 isomer	$C_{47}H_{80}O_{19}$	948.5294	L15	1.69
3.35	817.498	817.4980[M-H]⁻ 655.4273[M-H-Glc]⁻ 493.3653[M-H-2Glc]⁻	20(S)- Rf_3 isomer	$C_{42}H_{74}O_{15}$	818.5028	L15、Y5、N5	-3.67

3.38	815.4816	815.4802[M-H]⁻ 653.4259[M-H-Glc]⁻ 491.3736[M-H-2Glc] 391.2856	Panajaponol A/isomer	$C_{42}H_{72}O_{15}$	816.4871	L5、L15、Y5、N5	-2.82
3.67	1123.5924	1123.5951[M-H]⁻ 961.5277[M-H-Glc]⁻ 799.4742[M-H-2Glc]⁻ 637.4282[M-H-3Glc]⁻ 475.3876[M-H-4Glc]⁻	Notoginsenoside Fh_7 isomer	$C_{54}H_{92}O_{24}$	1124.5979	L5、L15、Y5、N5	-2.05
3.68	961.5383	961.5401[M-H]⁻ 799.4852[M-H-Glc]⁻	G-Re_3	$C_{48}H_{82}O_{19}$	962.545	L5、L15、Y5、N5	-1.14
3.82	831.4767	831.4831[M+FA-H]⁻ 785.4672[M-H]⁻ 653.4260[M-H-Xyl/Ara]⁻ 491.3778[M-H-Xyl/Ara-Glc]⁻	M-R_2/isomer	$C_{41}H_{70}O_{14}$	786.4766	L15、Y5、N5	2.04
3.97	1093.5796	1093.5849[M-H]⁻ 961.5383[M-H-Xyl/Ara]⁻ 799.5146[M-H-Xyl/Ara-Glc]⁻ 637.4307[M-H-Xyl/Ara-2Glc]⁻ 475.3716[M-H-Xyl/Ara-3Glc]⁻	20S-sanchirhinosides A_6 isomer	$C_{53}H_{90}O_{23}$	1094.5873	L15、Y5	-0.09
4.15	787.4832	787.4909[M-H]⁻ 655.4474[M-H-Xyl/Ara]⁻ 493.3920[M-H-Xyl/Ara-Glc]⁻	3β,12β,20,25-tetrahydroxydammArane-6-O-β-D-Xylopyranosyl-(1→2)-β-D-glucopyranoside isomer	$C_{41}H_{72}O_{14}$	788.4922	L15	1.52
4.48	961.538	961.5409[M-H]⁻ 799.4851[M-H-Glc]⁻ 781.4779[M-H-Glc-H_2O]⁻ 637.4334[M-H-2Glc-] 475.3780[M-H-3Glc]⁻	Re_1 isomer	$C_{48}H_{82}O_{19}$	962.545	L5、L15、Y5、N5	-0.83
4.61	1007.5436	1007.5537[M+FA-H]⁻ 961.5420[M-H]⁻ 799.4781[M-H-Glc]⁻	20-glu-G-Rf	$C_{48}H_{82}O_{19}$	962.545	L5、L15、Y5、N5	-4.99
4.64	931.5284	931.5332[M-H]⁻ 799.4894[M-H-Ara(f)]⁻ 637.4357[M-H-Ara(f)-Glc]⁻ 475.3795[M-H-Ara(f)-2Glc]⁻	G-Re_4	$C_{47}H_{80}O_{18}$	932.5345	L5、L15、Y5、N5	-1.82
4.66	845.4894	845.5101[M+FA-H]⁻ 799.4923[M-H]⁻ 653.4353[M-H-Rha]⁻ 491.3664[M-H-Rha-Glc]⁻	PG-F_{11} isomer	$C_{42}H_{72}O_{14}$	800.4922	L5、L15、Y5、N5	-9.88

4.68	831.475	831.4847[M+FA-H]$^-$ 785.4700[M-H]$^-$ 653.4272[M-H-Ara/Xyl]$^-$ 635.4084[M-H-Ara/Xyl-H_2O]$^-$ 491.3704[M-H-Ara/Xyl-Glc]$^-$	M-R_2/isomer	$C_{41}H_{70}O_{14}$	786.4766	L5、L15、Y5、N5	-1.53
4.73	1007.5446	1007.5449[M+FA-H]$^-$ 961.5403[M-H]$^-$ 799.4850[M-H-Glc]$^-$ 637.4181[M-H-2Glc]$^-$	G-Re_2	$C_{48}H_{82}O_{19}$	962.545	L5、L15、Y5、N5	-3.22
4.82	1077.5826	1077.5888[M-H]$^-$ 945.5443[M-H-Xyl/Ara]$^-$ 799.4837[M-H-Xyl/Ara-Rha]$^-$ 783.4936[M-H-Xyl/Ara-Glc]$^-$ 637.4249[M-H-Rha-Xyl/Ara-Glc]$^-$ 475.3740[M-H-Rha-Xyl/Ara-2Glc]$^-$	Floralginsenoside M isomer	$C_{53}H_{90}O_{22}$	1078.5924	L5、L15、Y5、N5	1.86
4.85	1093.5789	1093.5856[M-H]$^-$ 931.5350[M-H-Glc]$^-$ 799.4631[M-H-Glc-Xyl/Ara]$^-$ 637.4243[M-H-Xyl/Ara-2Glc]$^-$ 475.3803[M-H-3Glc-Xyl/Ara]$^-$	20S-sanchirhinosides A_6 isomer	$C_{53}H_{90}O_{23}$	1094.5873	L5、L15、Y5	0.55
4.9	883.5063	883.5079[M-H]$^-$ 841.4887[M-H-Ac]$^-$ 799.4767[M-H-2Ac]$^-$ 781.4664[M-H-2Ac-H_2O]$^-$ 763.4465[M-H-2Ac-2H_2O]$^-$ 637.4242[M-H-2Ac-Glc]$^-$ 475.3739[M-H-2Ac-2Glc]$^-$	6',6''-di-O-acetyl Rg_1 isomer	$C_{46}H_{76}O_{16}$	884.5133	L5、L15、Y5、N5	-0.91
4.91	861.4853	861.4935[M+FA-H]$^-$ 815.4784[M-H]$^-$ 653.4239[M-H-Glc]$^-$ 635.4399[M-H-Glc-H_2O]$^-$ 491.3707[M-H-2Glc]$^-$	Panajaponol A/isomer	$C_{42}H_{72}O_{15}$	816.4871	L5、L15、Y5、N5	1.10
5.04	653.4263	653.4280[M-H]$^-$ 491.3788[M-Glc]$^-$	PG-RT_5 isomer	$C_{36}H_{62}O_{10}$	654.4343	L5、L15、Y5、N5	0.31
5.19	1107.5973	1107.5999[M-H]$^-$ 961.5520[M-H-Rha]$^-$ 945.5238[M-H-Glc]$^-$ 927.4901[M-H-Glc-H_2O]$^-$ 783.5103[M-H-2Glc]$^-$ 637.4377[M-H-2Glc-Rha]$^-$ 475.3802[M-H-3Glc-Rha]$^-$	Yesanchinoside E/isomer	$C_{54}H_{92}O_{23}$	1108.6029	L5	-1.99
5.19	931.5284	977.5361[M+HCOO]$^-$ 931.5323[M-H]$^-$ 799.4895[M-H-Xyl]$^-$ 637.4340[M-H-Xyl-Glc]$^-$	NG-R_1	$C_{47}H_{80}O_{18}$	932.5345	L5、L15、Y5、N5	-1.82
5.22	1077.586	1077.5891[M-H]$^-$ 945.5427[M-H-Ara/Xyl]$^-$ 931.5314[M-H-Rha]$^-$ 799.5007[M-H-Rha-Ara/Xyl]$^-$ 637.4326[M-H-Rha-Xyl/Ara-Glc]$^-$ 475.3805[M-H-Rha-Xyl/Ara-2Glc]$^-$	Floralginsenoside M isomer	$C_{53}H_{90}O_{22}$	1078.5924	L5、L15、Y5、N5	-1.30

5.28	801.4938	801.4938[M-H]⁻ 655.4284[M-H-Rha]⁻ 493.3736[M-H-Rha-Glc]⁻	20(R)-G-Rf_2 isomer	$C_{42}H_{74}O_{14}$	802.5079	N5	7.86
5.3	799.4853	799.4887[M-H]⁻ 653.4289[M-H-Rha]⁻ 635.4220[M-H-Rha-H_2O]⁻ 491.3738[M-H-Rha-Glc]⁻	PG-F_{11} isomer	$C_{42}H_{72}O_{14}$	800.4922	L5、L15、N5	-1.13
5.34	961.5397	961.5439[M-H]⁻ 799.4901[M-H-Glc]⁻ 637.4361[M-H-2Glc] 475.3979[M-H-3Glc]	G-Re_1	$C_{48}H_{82}O_{19}$	962.545	L5、L15、Y5、N5	-2.60
5.35	1003.5427	1003.5500[M-H]⁻ 961.5477[M-H-Ac]⁻ 799.4829[M-H-Ac-Glc]⁻ 637.4480[M-H-Ac-Glc-Glc]⁻ 475.3933[M-H-Ac-Glc-Glc-Glc]	6-acetyl ginsenoside-Rg_3/isomer	$C_{50}H_{84}O_{20}$	1004.5556	L5、L15、Y5、N5	5.08
5.44	885.482	885.4974[M-H]⁻ 841.4966[M-H-CO_2]⁻ 799.4868[M-H-CO_2-Ac]⁻ 679.4388[M-H-CO_2-Glc]⁻ 637.4320[M-H-CO_2-Ac-Glc]⁻ 619.4206[M-H-CO_2-Ac-Glc-H_2O]⁻ 475.3780[M-H-CO_2-Ac-2Glc]⁻	mRg_1 isomer	$C_{45}H_{74}O_{17}$	886.4926	L5、L15、Y5、N5	3.16
5.46	861.4864	861.4869[M+FA-H]⁻ 815.4838[M-H]⁻ 653.4208[M-H-Glc]⁻ 491.3700[M-H-2Glc]⁻	Panajaponol A/isomer	$C_{42}H_{72}O_{15}$	816.4871	L5、L15、Y5、N5	-5.52
5.47	841.497	841.4979[M-H]⁻ 799.4883[M-H-Ac]⁻ 679.4424[M-H-Glc]⁻ 637.4333[M-H-Ac-Glc]⁻ 619.4212[M-H-Ac-Glc-H_2O]⁻ 571.4000 475.3784[M-H-Ac-2Glc]⁻	noto-Rt/isomer	$C_{44}H_{74}O_{15}$	842.5028	L5、L15、Y5、N5	-2.38
5.54	931.5264	931.5268[M-H]⁻ 799.4871[M-H-Xyl/Ara]⁻ 637.4097[M-H-Xyl/Ara-Glc]⁻ 637.4328[M-H-Xyl/Ara-Glc]⁻ 475.3707 [M-H-Xyl/Ara-2Glc]⁻	Re4 isomer/NG-R_1 isomer	$C_{47}H_{80}O_{18}$	932.5345	L5、L15、Y5、N5	0.32
5.6	1107.5946	1107.5993[M-H]⁻ 945.5454[M-H-Glc]⁻ 799.4826[M-H-Glc-Rha]⁻ 637.4536[M-H-Glc-Rha-Glc]⁻ 475.3969[M-H-Glc-Glc-Glc-Rha]	Yesanchinoside E/isomer	$C_{54}H_{92}O_{23}$	1108.6029	L5、L15、N5	0.45
5.76	883.5036	883.5039[M-H]⁻ 841.4901[M-H-Ac]⁻ 799.4997[M-H-2Ac]⁻ 637.4221[M-H-2Ac-Glc]⁻ 475.3955[M-H-2Ac-2Glc]⁻	6',6''-di-O-acetyl Rg_1 isomer	$C_{46}H_{76}O_{16}$	884.5133	L5、L15	2.15

5.77	845.493	845.4931[M+HCOO]⁻ 799.4878[M-H]⁻ 637.4344[M-H-Glc]⁻ 475.3796[M-H-2Glc]⁻	G-Rg_1	$C_{42}H_{72}O_{14}$	800.4922	L5、L15、Y5、N5	-4.25
5.81	991.5497	991.5509[M+HCOO]⁻ 945.5451[M-H]⁻ 783.4931[M-H-Glc]⁻ 637.4339[M-H-Glc-Rha]⁻	G-Re	$C_{48}H_{82}O_{18}$	946.5501	L5、L15、Y5、N5	-2.96
5.92	811.4851	811.4875[M-H]⁻ 769.4769[M-H-C_2H_2O]⁻ 637.4332[M-H-C_2H_2O-Ara]⁻ 475.3822[M-H-C_2H_2O-Ara-Glc]⁻ 457.3785[M-H-C_2H_2O-Ara-Glc-H_2O]⁻	β-D-Glucopyranoside, (3β, 6α,12β,20*R*)-3,12,20-trihydroxydammar-24-en-6-yl 2-*O*-β-D-Xylopyranosyl-, 6-acetate isomer	$C_{43}H_{72}O_{14}$	812.4922	L5、L15、Y5、N5	-0.86
6.03	841.4946	841.4992[M-H]⁻ 799.4870[M-H-Ac]⁻ 781.4783[M-H-Ac-H_2O]⁻ 637.4433[M-H-Ac-Glc]⁻ 571.3910 475.3696[M-H-Ac-2Glc]⁻	noto-Rt/isomer	$C_{44}H_{74}O_{15}$	842.5028	L5、L15、Y5、N5	0.48
6.07	931.5273	931.5295[M-H]⁻ 799.4904[M-H-Ara/Xyl]⁻ 637.4383[M-H-Ara/Xyl-Glc]⁻ 475.3807[M-H-Ara/Xyl-2Glc]⁻	Re4 isomer/NG-R_1 isomer	$C_{47}H_{80}O_{18}$	932.5345	L5、L15	-0.64
6.09	769.4723	769.4764[M-H]⁻ 637.4358[M-H-Xyl/Ara]⁻ 475.3834 [M-H-Xyl/Ara-Glc]⁻	F_3 isomer	$C_{41}H_{70}O_{13}$	770.4816	L15、Y5	1.95
6.12	885.4868	885.4981[M-H]⁻ 841.4957[M-H-CO_2]⁻ 799.4857[M-H-CO_2-Ac]⁻ 781.4785[M-H-CO_2-Ac-H_2O]⁻ 637.4350[M-H-CO_2-Ac-Glc]⁻ 475.3668[M-H-CO_2-Ac-2Glc]⁻	mRg_1 isomer	$C_{45}H_{74}O_{17}$	886.4926	L5、L15、Y5	-2.26
6.13	825.496	825.5019[M-H]⁻ 783.4912[M-H-Ac]⁻ 765.4817[M-H-Ac-H_2O]⁻ 621.4336[M-H-Ac-Glc]⁻ 459.3839[M-H-Ac-2Glc]⁻	Rs_3 isomer	$C_{44}H_{74}O_{14}$	826.5079	L5、L15、Y5、N5	4.97
6.14	1031.5427	1031.5427[M-H]⁻ 989.5603[M-H-Ac]⁻ 945.5464[M-H-Ac-CO_2]⁻ 799.4866[M-H-Ac-Rha]⁻ 637.4181[M-H-Ac-Rha-Glc]⁻ 475.3799[M-H-Ac-CO_2-Rha-2Glc]⁻	mRd isomer	$C_{51}H_{84}O_{21}$	1032.5505	L15、Y5	0.00
6.15	831.4744	831.4778[M+FA-H]⁻ 785.4726[M-H]⁻ 653.4287[M-H-Xyl/Ara]⁻ 491.3841[M-H-Xyl/Ara-Glc]⁻	M-R_2/isomer	$C_{41}H_{70}O_{14}$	786.4766	L5、L15	-4.84

6.16	969.5454	969.5454[M-H-H_2O]$^-$ 927.5341[M-H-H_2O-Ac]$^-$ 909.5226[M-H-2H_2O-Ac]$^-$ 765.4705[M-H-H_2O-Ac-Glc]$^-$ 621.4331[M-H-Ac-2Glc]$^-$ 459.3891[M-H-Ac-3Glc]$^-$	6'''-O-acetylgypenoside XVII isomer	$C_{50}H_{84}O_{19}$	988.5607	L5、L15、Y5、N5	-3.14
6.18	1123.5938	1123.5953[M-H]$^-$ 961.5364[M-H-Glc]$^-$ 799.4912[M-H-2Glc]$^-$ 637.4347[M-H-3Glc]$^-$ 475.3801[M-H-4Glc]$^-$	Notoginsenoside Fh_7 isomer	$C_{54}H_{92}O_{24}$	1124.5979	L5、L15、Y5、N5	-3.29
6.19	825.4991	825.5018[M-H]$^-$ 783.495[M-H-Ac]$^-$ 765.4748[M-H-Ac-H_2O]$^-$ 679.4401[M-H-Rha]$^-$ 637.4332[M-H-Ac-Rha]$^-$ 619.4226[M-H-Ac-Rha-H_2O]$^-$ 571.4032 475.3756[M-H-Ac-Rha-Glc]$^-$	β-D-Glucopyranoside, (3β, 6α,12β,20*R*)-3,12,20-trihydroxydammar-24-en-6-yl 2-*O*-(6-deoxy-α-L-mannopyranosyl)-, 6-acetate isomer	$C_{44}H_{74}O_{14}$	826.5079	L5、L15、N5	1.21
6.2	799.4847	799.4860[M-H]$^-$ 637.4330[M-H-Glc]$^-$ 475.3830[M-H-2Glc]$^-$	Rg_1 isomer	$C_{42}H_{72}O_{14}$	800.4922	L5、L15、Y5	-0.38
6.26	1029.5618	1029.5673[M-H]$^-$ 987.5567[M-H-Ac]$^-$ 945.5432[M-H-2Ac]$^-$ 783.5020[M-H-Ac-Ac-Glc]$^-$ 621.4281[M-H-Ac-Ac-2Glc]$^-$ 459.3810[M-H-Ac-Ac-3Glc]$^-$	Diacetyl-G-Rd isomer	$C_{52}H_{86}O_{20}$	1030.5712	Y5	1.55
6.29	679.4419	679.4447[M-H]$^-$ 637.4311[M-H-Ac]$^-$ 619.4212[M-H-Ac-H_2O]$^-$ 475.3802[M-H-Ac-Glc]$^-$	20R-ginsenoside Rh1 6‘-acetate isomer	$C_{38}H_{64}O_{10}$	680.4499	L15、Y5、N5	0.29
6.29	955.4992	957.5016[M-H]$^-$ 956.4995[M-H]$^-$ 794.4441[M-H-Glc]$^-$	G-Ro	$C_{48}H_{76}O_{19}$	956.4981	L5、L15、Y5、N5	-9.30
6.37	845.4893	845.4843 799.4884 653.4308 491.3699	PG-F_{11} isomer	$C_{42}H_{72}O_{14}$	800.4922	L15、N5	-5.00
6.43	931.5232	931.5243[M-H]$^-$ 799.4716[M-H-Ara/Xyl]$^-$ 637.4242[M-H-Ara/Xyl-Glc]$^-$ 475.3887[M-H-Ara/Xyl-2Glc]$^-$	Re_4 isomer/NG-R_1 isomer	$C_{47}H_{80}O_{18}$	932.5345	L5、L15	3.76

6.51	811.4834	811.4895[M-H]⁻ 769.4773[M-H-Ac]⁻ 637.4368[M-H-Ac-Ara/Xyl]⁻ 475.3959[M-H-Ac-Ara/Xyl-Glc]⁻	β-D-Glucopyranoside, (3β, 6α,12β,20*R*)-3,12,20-trihydroxydammar-24-en-6-yl 2-*O*-β-D-Xylopyranosyl-, 6-acetate isomer	$C_{43}H_{72}O_{14}$	812.4922	L5、L15、Y5	1.23
6.52	1093.5808	1093.5847[M-H]⁻ 961.5435[M-H-Xyl/Ara]⁻ 931.5202[M-H-Glc]⁻ 799.4910[M-H-Glc-Xyl/Ara]⁻ 637.4374[M-H-2Glc-Xyl/Ara]⁻ 475.3764[M-H-3Glc-Xyl/Ara]⁻	20S-sanchirhinosides A_6 isomer	$C_{53}H_{90}O_{23}$	1094.5873	L5、L15、Y5、N5	-1.19
6.54	1003.5454	1003.5481[M-H]⁻ 961.5396[M-H-Ac]⁻ 799.4832[M-H-Ac-Glc]⁻ 637.4175[M-H-Ac-Glc-Glc]⁻ 475.3772[M-H-Ac-Glc-Glc-Glc]	6-acetyl ginsenoside-Rg_3/isomer	$C_{50}H_{84}O_{20}$	1004.5556	L15、Y5、N5	2.39
6.54	769.4724	769.4772[M-H]⁻ 637.4407[M-H-Xyl/Ara]⁻ 475.3722[M-H-Xyl/Ara-Glc]⁻	F_3 isomer	$C_{41}H_{70}O_{13}$	770.4816	L15、Y5	1.82
6.64	947.522	947.5277[M+FA-H]⁻ 901.5203[M-H]⁻ 769.4726[M-H-Xyl/Ara]⁻ 637.4319[M-H-2Xyl/Ara]⁻ 475.3788[M-H-2Xyl/Ara-Glc]⁻	chikusetsusaponin LM_2 isomer	$C_{46}H_{78}O_{17}$	902.5239	L15	-4.66
6.74	925.4818	925.4842[M-H]⁻	CS-IV	$C_{47}H_{74}O_{18}$	926.4875	L5、L15、Y5、N5	-2.27
6.81	1153.6172	1153.6211[M+FA-H]⁻ 1107.5999[M-H]⁻ 945.5462[M-H-Glc]⁻ 783.4902[M-H-Glc-Glc]⁻ 621.4482[M-H-Glc-Glc-Glc] 459.3881[M-H-Glc-Glc-Glc-Glc]	Rb_1 isomer	$C_{54}H_{92}O_{23}$	1108.6029	L5、L15、Y5、N5	-4.33
6.95	825.5009	825.4978[M-H]⁻ 783.4903[M-H-Ac]⁻ 637.4395[M-H-Ac-Rha]⁻ 475.3888[M-H-Ac-Rha-Glc]⁻	β-D-Glucopyranoside, (3β, 6α,12β,20*R*)-3,12,20-trihydroxydammar-24-en-6-yl 2-*O*-(6-deoxy-α-	$C_{44}H_{74}O_{14}$	826.5079	Y5、N5	-0.97

			L-mannopyranosyl)-, 6-acetate isomer				
6.98	1149.6074	1149.6056[M-H]⁻ 1107.5958[M-H-Ac]⁻ 1089.5855[M-H-Ac-H_2O]⁻ 987.5480[M-H-Glc]⁻ 945.5435[M-H-Ac-Glc]⁻ 927.5342[M-H-Ac-Glc-H2O]⁻ 783.4877[M-H-Ac-2Glc] 621.4405[M-H-Ac-3Glc]⁻ 459.3881[M-H-Ac-4Glc]⁻	Q-R_1 isomer	$C_{56}H_{94}O_{24}$	1150.6135	L5、L15、Y5、N5	-1.48
7.04	769.473	769.4780[M-H]⁻ 637.4293[M-H-Ara/Xyl]⁻ 619.4254[M-H-Ara/Xyl-H_2O]⁻ 475.3819[M-H-Ara/Xyl-Glc]⁻	F_3 isomer	$C_{41}H_{70}O_{13}$	770.4816	L5、L15、Y5、N5	1.04
7.08	1119.5959	1119.5964 1077.5883 1059.5768	R-s_2 isomer	$C_{55}H_{92}O_{23}$	1120.6029	L5、L15、Y5、N5	-0.71
7.13	1077.5817	1077.5881[M-H]⁻ 945.5424[M-H-Xyl/Ara]⁻ 783.4820[M-H-Xyl/Ara-Glc]⁻ 621.4493[M-H-Xyl/Ara-2Glc]⁻ 459.3929[M-H-Xyl/Ara-3Glc]⁻	Rb_2 isomer	$C_{53}H_{90}O_{22}$	1078.5924	L5、L15、Y5、N5	2.69
7.13	793.4396	793.4404[M-H]⁻ 631.3859[M-H-Glc]⁻ 613.3744[M-H-Glc-H_2O]⁻ 569.3856[M-H-Glc-CO_2]⁻ 455.3530[M-H-Glc-GlcA]⁻	CS-Iva	$C_{42}H_{66}O_{14}$	794.4453	L5、L15、Y5、N5	-2.65
7.17	915.5306	915.5325[M-H]⁻ 783.4918[M-H-Xyl/Ara]⁻ 637.4339[M-H-Xyl/Ara-Rha]⁻ 475.3944[M-H-Xyl/Ara-Rha-Glc]⁻	β-D-Glucopyranoside, (3β, 6α,12β)-3,12-dihydroxy-20-(β-D-Xylopyranosyloxy) dammar-24-en-6-yl 2-*O*-(6-deoxy-α-L-mannopyranosyl)-isomer	$C_{47}H_{80}O_{17}$	916.5396	L5、L15、Y5、N5	1.31

7.27	679.4408	679.4489[M-Ac]$^-$ 637.4302[M-H]$^-$ 475.3788[M-H-Glc]$^-$	20R-ginsenoside Rh1 6'-acetate isomer	$C_{38}H_{64}O_{10}$	680.4499	L15、Y5、N5	1.91
7.36	841.4969	841.5016[M-H]$^-$ 805.4004[M-H-H2O]$^-$ 799.4845[M-H-Ac]$^-$ 781.4655[M-H-Ac-H2O]$^-$ 637.4320[M-H-Ac-Glc]$^-$ 475.3926[M-H-Ac-2Glc]$^-$	noto-Rt/isomer	$C_{44}H_{74}O_{15}$	842.5028	L5、L15、Y5、N5	-2.26
7.41	1119.5978	1119.5997[M-H]$^-$ 1077.5891[M-H-Ac]$^-$ 1059.5795[M-H-Ac-H_2O]$^-$ 945.5502[M-H-Ac-Xyl/Ara]$^-$ 783.4929[M-H-Ac-Xyl/Ara-Glc]$^-$ 765.4853[M-H-Ac-Xyl/Ara-Glc-H_2O]$^-$ 621.4379[M-H-Ac-Xyl/Ara-2Glc]$^-$ 459.3871[M-H-Ac-Xyl/Ara-3Glc]$^-$	Rs_1 isomer	$C_{55}H_{92}O_{23}$	1120.6029	L5、L15、Y5、N5	-2.41
7.48	1163.589	1163.5697[M-H]$^-$ 1119.5992[M-HCOO]$^-$ 1077.5875[M-H-HCOO-Ac]$^-$ 1059.5772[M-H-HCOO-Ac-H_2O]$^-$ 945.5770[M-H-HCOO-Ac-Xyl/Ara]$^-$ 783.4948[M-H-HCOO-Ac-Xyl/Ara-Glc]$^-$ 765.4912[M-H-HCOO-Ac-Xyl/Ara-Glc-H_2O]$^-$ 621.4249[M-H-HCOO-Ac-Xyl/Ara-2Glc]$^-$ 459.3936[M-H-HCOO-Ac-Xyl/Ara-3Glc]$^-$	Malonyl-ginsenosideRc isomer	$C_{56}H_{92}O_{25}$	1164.5928	L5、L15、Y5、N5	-3.44
7.49	1059.5786	1059.5801 945.5463 783.5000 621.4579	Quinquenoside I isomer	$C_{52}H_{86}O_{19}$	1014.5763	L15、Y5、N5	-4.54
7.52	1149.6075	1149.6048 1107.5995 1089.5870 1077.5993 987.5319 945.5390 783.4874 621.4255	Q-R_1 isomer	$C_{56}H_{94}O_{24}$	1150.6135	L5、L15、N5	-1.57
7.54	1077.5881	1077.5852[M-H]$^-$ 945.5452[M-H-Xyl/Ara]$^-$ 915.5419[M-H-Glc]$^-$ 783.4926[M-H-Glc-Xyl/Ara]$^-$ 765.4782[M-H-Glc-Xyl/Ara-H_2O]$^-$ 621.4221[M-H-2Glc-Xyl/Ara]$^-$ 459.3994[M-H-3Glc-Xyl/Ara]$^-$	Rb_2 isomer	$C_{53}H_{90}O_{22}$	1078.5924	L5、L15、Y5、N5	-3.25

7.65	931.5248	931.5227[M-H]$^-$ 799.4649[M-H-Xyl/Ara]$^-$ 781.4975[M-H-Xyl/Ara-H_2O]$^-$ 637.3980[M-H-Xyl/Ara-Glc]$^-$ 475.3746[M-H-Xyl/Ara-2Glc]$^-$	Re_4 isomer/NG-R_1 isomer	$C_{47}H_{80}O_{18}$	932.5345	L15、Y5、N5	2.04
7.69	829.4938	829.4938[M+FA-H]$^-$ 783.4847[M-H]$^-$ 621.4377[M-H-Glc]$^-$ 475.3752[M-H-Glc-Rha]$^-$	Rg_2 isomer	$C_{42}H_{72}O_{13}$	784.4973	L5、L15、Y5、N5	6.13
7.71	961.5355	961.5419[M-H]$^-$ 799.4892[M-H-Glc]$^-$ 637.4323[M-H-2Glc]$^-$ 475.3792[M-H-3Glc]$^-$	Re_1 isomer	$C_{48}H_{82}O_{19}$	962.545	L5、L15、Y5、N5	1.77
7.76	679.4408	679.4396[M-Ac]$^-$ 637.4322[M-H]$^-$ 619.4113[M-H-H_2O]$^-$ 475.3744[M-H-Ac-Glc]$^-$	20R-ginsenoside Rh1 6‘-acetate isomer	$C_{38}H_{64}O_{10}$	680.4499	L15、Y5、N5	1.91
7.8	815.479	815.4803[M-H]$^-$ 653.4264[M-H-Glc]$^-$ 491.3828[M-H-Glc-Glc]$^-$	Panajaponol A/isomer	$C_{42}H_{72}O_{15}$	816.4871	L5、L15、Y5、N5	0.37
7.97	1119.5946	1119.5988[M-H]$^-$ 1077.5883[M-H-Ac]$^-$ 1059.5782[M-H-Ac-H_2O]$^-$ 945.5323[M-H-Ac-Xyl/Ara]$^-$ 783.4965[M-H-Ac-Xyl/Ara-Glc]$^-$ 621.4344[M-H-Ac-Xyl/Ara-2Glc]$^-$ 459.3851[M-H-Ac-Xyl/Ara-3Glc]$^-$	Rs_1 isomer	$C_{55}H_{92}O_{23}$	1120.6029	L5、L15、Y5、N5	0.45
7.97	1059.5706	1059.5786[M+FA-H]$^-$ 945.5333[M-H-C_4H_4O]$^-$ 927.5494[M-H-C_4H_4O-H_2O]$^-$ 765.4869[M-H-C_4H_4O-Glc-H_2O]$^-$ 621.4231[M-H-C_4H_4O-2Glc]$^-$ 459.3881[M-H-C_4H_4O-3Glc]$^-$	Quinquenoside I isomer	$C_{52}H_{86}O_{19}$	1014.5763	L15、N5	3.35
8.09	987.5542	987.5578[M-H]$^-$ 945.5469[M-H-Ac]$^-$ 927.5306[M-H-Ac-H_2O]$^-$ 841.4994[M-H-Rha]$^-$ 799.5353[M-H-Ac-Rha]$^-$ 637.4142[M-H-Ac-Rha-Glc]$^-$ 475.3844[M-H-Ac-Rha-Glc-Glc]$^-$	Pseudoginsenoside Rs_1/isomer	$C_{50}H_{84}O_{19}$	988.5607	L5、L15、Y5、N5	-1.32
8.19	807.4815	807.4815[M-H]$^-$ 765.4404[M-H-Ac]$^-$ 603.3835[M-H-Ac-Glc]$^-$ 441.3430[M-H-Ac-2Glc]$^-$	20Z-ginsenoside-Rs_4 isomer	$C_{44}H_{72}O_{13}$	808.4973	L5、L15、Y5、N5	9.91
8.25	841.4976	841.4957[M-H]$^-$ 799.4828[M-H-Ac]$^-$ 781.4806[M-H-Ac-H_2O]$^-$ 679.4316[M-H-Glc]$^-$ 637.4337[M-H-Ac-Glc]$^-$ 619.4211[M-H-Ac-Glc-H_2O]$^-$ 475.3762[M-H-Ac-2Glc]$^-$	noto-Rt/isomer	$C_{44}H_{74}O_{15}$	842.5028	L5、L15、Y5、N5	-3.09

8.31	945.5443	945.5464[M-H]$^-$ 783.4924[M-H-Glc]$^-$ 621.4346[M-H-2Glc]$^-$ 459.3801[M-H-3Glc]$^-$	Rd isomer	$C_{48}H_{82}O_{18}$	946.5501	L5、L15、Y5、N5	-2.12
8.34	829.4948	829.4947[M+FA-H]$^-$ 783.4879[M-H]$^-$ 637.4256[M-H-Rha]$^-$ 619.4134[M-H-Rha-H_2O]$^-$ 475.3664[M-H-Rha-Glc]$^-$	Rg_2 isomer	$C_{42}H_{72}O_{13}$	784.4973	L5、L15、Y5、N5	2.04
8.4	987.5528	987.5528[M-H]$^-$ 945.5436[M-H-Ac]$^-$ 927.5336[M-H-Ac-H_2O]$^-$ 765.4803[M-H-Ac-H_2O-Glc]$^-$ 621.4381[M-H-Ac-2Glc]$^-$ 459.3827[M-H-Ac-3Glc]$^-$	6'''-O-acetylgypenoside XVII isomer	$C_{50}H_{84}O_{19}$	988.5607	L5、L15、Y5、N5	0.10
8.6	1031.546	1031.5472[M-H]$^-$ 987.5576[M-H-CO_2]$^-$ 945.5482[M-H-CO_2-Ac]$^-$ 927.5375[M-H-CO_2-Ac-H_2O]$^-$ 783.4942[M-H-CO_2-Ac-Glc]$^-$ 765.4810[M-H-CO_2-Ac-Glc-H_2O]$^-$ 621.4350[M-H-CO_2-Ac-2Glc]$^-$ 459.3804[M-H-CO_2-Ac-3Glc]$^-$	Malonyl-ginsenoside Re isomer	$C_{51}H_{84}O_{21}$	1032.5505	L5、L15、Y5、N5	-3.20
8.69	783.4893	783.4907[M-H]$^-$ 621.4342[M-H-Glc]$^-$ 459.3858[M-H-2Glc]$^-$	20(S)-G-Rg_3 isomer	$C_{42}H_{72}O_{13}$	784.4973	N5	0.26
8.75	961.5357	961.5382[M-H]$^-$ 799.4880[M-H-Glc]$^-$ 781.4725[M-H-Glc-H_2O]$^-$ 679.4304 637.4469[M-H-2Glc]$^-$ 475.3603[M-H-3Glc]$^-$	Re_1 isomer	$C_{48}H_{82}O_{19}$	962.545	L5、L15、Y5、N5	1.56
8.77	831.4737	831.4722[M+FA-H]$^-$ 785.4706[M-H]$^-$ 653.4274[M-H-Xyl/Ara]$^-$ 491.3850[M-H-Xyl/Ara-Glc]$^-$	M-R_2/isomer	$C_{41}H_{70}O_{14}$	786.4766	L5、L15、Y5、N5	-2.29
8.9	977.5219	977.5219[M+FA-H]$^-$ 931.5304[M-H]$^-$ 799.4721[M-H-Xyl/Ara]$^-$ 637.4305[M-H-Xyl/Ara-Glc]$^-$ 475.3873[M-H-Xyl/Ara-2Glc]$^-$	Re_4 isomer/NG-R_1 isomer	$C_{47}H_{80}O_{18}$	932.5345	L5、L15、Y5、N5	6.76
8.98	913.5198	913.5173[M+FA-H]$^-$ 867.5149[M-H]$^-$ 781.4694[M-H-C_4H_4O-H_2O]$^-$ 799.4615[M-H-C_4H_4O]$^-$ 637.4379[M-H-C_4H_4O-Glc]$^-$ 619.4215[M-H-C_4H_4O-Glc-H_2O]$^-$ 475.38249[M-H-C_4H_4O-2Glc]$^-$	koryoginsenoside-R_1/isomer	$C_{46}H_{76}O_{15}$	868.5184	L5、L15、Y5、N5	-4.96
9.06	1031.548	1031.5461[M-H]$^-$ 987.5568[M-H-CO_2]$^-$ 945.5492[M-H-CO_2-Ac]$^-$ 783.4991[M-H-CO_2-Ac-Glc]$^-$ 621.4164[M-H-CO_2-Ac-Glc-Glc]$^-$ 459.3901[M-H-CO_2-Ac-Glc-Glc-Glc]$^-$	Malonyl-ginsenoside Re isomer	$C_{51}H_{84}O_{21}$	1032.5505	L5、L15、Y5、N5	-5.14

9.06	987.553	987.5548[M-H]⁻ 945.5493[M-H-Ac]⁻ 927.5308[M-H-Ac-H_2O]⁻ 783.4851[M-H-Ac-Glc]⁻ 621.4411[M-H-Ac-Glc-Glc]⁻ 459.3694[M-H-Ac-Glc-Glc-Glc]⁻	6'''-O-acetylgypenoside XVII isomer	$C_{50}H_{84}O_{19}$	988.5607	L5、L15、Y5、N5	-0.10
9.08	1239.632	1107.5982[M-H-Xyl]⁻ 945.5493[M-H-Xyl-Glc]⁻	NG-R_4	$C_{55}H_{100}O_{30}$	1240.6299	L5、L15、Y5、N5	-7.99
9.09	845.4926	845.4900[M+HCOO]⁻ 799.4860[M-H]⁻ 637.4344[M-H-Glc]⁻ 475.3802[M-H-2Glc]⁻	20(S)-G-Rf-1a	$C_{42}H_{72}O_{14}$	800.4922	L5、L15、Y5、N5	-2.00
9.09	799.4869	799.4891[M-H]⁻ 637.4353[M-H-Glc]⁻ 475.3801[M-H-2Glc]⁻	20(S)-G-Rf	$C_{42}H_{72}O_{14}$	800.4922	L5、L15、Y5、N5	-3.13
9.09	799.4869	799.4891[M-H]⁻ 637.4353[M-H-Glc]⁻ 475.3801[M-H-2Glc]⁻	20(R)-G-Rf	$C_{42}H_{72}O_{14}$	800.4922	L5、L15、Y5、N5	-3.13
9.15	913.4741	913.4822[M+FA-H]⁻ 867.5110[M-H]⁻ 799.4850[M-H-C_4H_4O]⁻ 637.4326[M-H-C_4H_4O-Glc]⁻ 475.3892[M-H-C_4H_4O-2Glc]⁻	koryoginsenoside-R_1/isomer	$C_{46}H_{76}O_{15}$	868.5184	L5、L15、Y5、N5	-0.46
9.52	1269.65	1107.6022[M-H-Glc]⁻ 1089.5601[M-H-Glc-H_2O]⁻ 945.5489[M-H-Glc-Glc]⁻ 783.4800[M-H-Glc-Glc-Glc]⁻ 621.4350[M-H-Glc-Glc-Glc-Glc]⁻ 459.3861[M-H-Glc-Glc-Glc-Glc-Glc]	Ra_0 isomer	$C_{60}H_{102}O_{28}$	1270.6558	L5、L15、Y5、N5	-1.58
9.66	947.5235	947.5275[M+FA-H]⁻ 901.5202[M-H]⁻ 769.4802[M-H-Xyl/Ara]⁻ 637.4297[M-H-2Xyl/Ara]⁻ 475.3774[M-H-2Xyl/Ara-Glc]⁻	chikusetsusaponin LM_2 isomer	$C_{46}H_{78}O_{17}$	902.5239	L5、L15、Y5、N5	-4.55
9.67	845.4901	845.4864[M+FA-H]⁻ 799.4923[M-H]⁻ 637.4226[M-H-Glc]⁻ 475.3758[M-H-2Glc]⁻	Rg_1 isomer	$C_{42}H_{72}O_{14}$	800.4922	L15、Y5、N5	-9.88
9.75	945.5404	945.5440[M-H]⁻ 783.4906[M-H-Glc]⁻ 621.4374[M-H-2Glc]⁻ 459.3732[M-H-3Glc]⁻	Rd isomer	$C_{48}H_{82}O_{18}$	946.5501	L5、L15、Y5、N5	2.01
9.77	1031.5434	1031.5494[M-H]⁻ 987.5551[M-H-CO_2]⁻ 945.5428[M-H-CO_2-Ac]⁻ 783.5040[M-H-CO_2-Ac-Glc]⁻ 621.4093[M-H-CO_2-Ac-2Glc]⁻ 459.3853[M-H-CO_2-Ac-3Glc]⁻	Malonyl-ginsenoside Re isomer	$C_{51}H_{84}O_{21}$	1032.5505	L15、Y5、N5	-0.68

9.78	987.5535	987.5529[M-H]⁻ 945.5419[M-H-Ac]⁻ 927.5268[M-H-Ac-H_2O]⁻ 783.4873[M-H-Ac-Glc]⁻ 765.4957[M-H-Ac-Glc-H_2O]⁻ 621.4374[M-H-Ac-2Glc]⁻ 459.3875[M-H-Ac-3Glc]	6'''-O-acetylgypenoside XVII isomer	$C_{50}H_{84}O_{19}$	988.5607	L5、L15、Y5、N5	-0.61
9.83	815.4818	815.4829[M+HCOO]⁻ 769.4762[M-H]⁻ 637.4333[M-H-Xyl]⁻ 475.3792[M-H-Xyl-Glc]⁻	20(S)-NG-R_2	$C_{41}H_{70}O_{13}$	770.4816	L5、L15、Y5、N5	-3.12
9.83	769.4761	769.4793[M-H]⁻ 637.4369[M-H-Ara]⁻ 475.3803[M-H-Ara-Glc]⁻	G-F_3	$C_{41}H_{70}O_{13}$	770.4816	L5、L15、Y5、N5	-2.99
10.25	1209.6298	1077.5890[M-H-Xyl]⁻ 945.5514[M-H-Xyl-Ara(f)]⁻ 783.4951[M-H-Xyl-Ara(f)-Glc]⁻ 621.4350[M-H-Xyl-Ara(f)-2Glc]⁻	G-Ra_2	$C_{58}H_{98}O_{26}$	1210.6346	L5、L15、Y5、N5	-2.48
10.39	887.4989	887.5080[M+FA-H]⁻ 841.5007[M-H]⁻ 799.4905[M-H-Ac]⁻ 781.4728[M-H-Ac-H_2O]⁻ 637.4221[M-H-Ac-Glc]⁻ 475.3722[M-H-Ac-2Glc]⁻	noto-Rt/isomer	$C_{44}H_{74}O_{15}$	842.5028	L5、L15、Y5、N5	-6.77
10.42	1029.563	1029.5696[M-H]⁻ 987.5551[M-H-Ac]⁻ 945.5430[M-H-2Ac]⁻ 783.4938[M-H-2Ac-Glc]⁻ 621.4501[M-H-2Ac-2Glc]⁻ 459.4029[M-H-2Ac-3Glc]⁻	Diacetyl-G-Rd isomer	$C_{52}H_{86}O_{20}$	1030.5712	L15、Y5、N5	0.39
10.42	1239.6404	1107.5963[M-H-Xyl]⁻ 945.5535[M-H-Xyl-Glc]⁻ 783.4811[M-H-Xyl-2Glc]⁻	G-Ra_3	$C_{59}H_{100}O_{27}$	1240.6452	L5、L15、Y5、N5	-2.42
10.47	769.4748	769.4790[M-H]⁻ 637.4347[M-H-Xyl]⁻ 475.3819[M-H-Xyl-Glc]⁻	20(R)-NG-R_2	$C_{41}H_{70}O_{13}$	770.4816	L5、L15、Y5	-1.30
10.49	1107.5974	1107.6017[M-H]⁻	G-Rb_1	$C_{54}H_{92}O_{23}$	1108.6029	L5、L15、Y5、N5	-2.08
10.55	783.4924	783.4928[M-H]⁻ 637.4318[M-H-Rha]⁻ 475.3794[M-H-Rha-Glc]⁻	20(S)-G-Rg_2	$C_{42}H_{72}O_{13}$	784.4973	L5、L15、Y5、N5	-3.70

10.55	783.4924	783.4928[M-H]$^-$ 637.4318[M-H-Rha]$^-$ 475.3794[M-H-Rha-Glc]$^-$	20(R)-G-Rg_2	$C_{42}H_{72}O_{13}$	784.4973	L5、L15、Y5、N5	-3.70
10.64	913.5193	913.5207[M+FA-H]$^-$ 867.5122[M-H]$^-$ 799.4863[M-H-C_4H_4O]$^-$ 781.4766[M-H-C_4H_4O-H_2O]$^-$ 637.4431[M-H-C_4H_4O-Glc]$^-$ 619.4244[M-H-C_4H_4O-Glc-H_2O]$^-$ 475.3763[M-H-C_4H_4O-2Glc]$^-$	koryoginsenoside-R_1/isomer	$C_{46}H_{76}O_{15}$	868.5184	L5、L15、Y5、N5	-1.84
10.64	683.4398	683.4405[M+FA-H]$^-$ 637.4328[M-H]$^-$ 475.3751[M-H-Glc]$^-$	F_1 isomer	$C_{36}H_{62}O_9$	638.4394	L5、L15、Y5、N5	-1.88
10.78	827.4786	827.4932[M+FA-H]$^-$ 781.4815[M-H]$^-$ 635.4420[M-H-Rha-Glc]$^-$ 473.3696[M-H-Rha-Glc]$^-$	ginsenoside Rh_{14} isomer	$C_{42}H_{70}O_{13}$	782.4816	N5	-9.85
10.84	987.5531	987.5546 945.5432 927.5393 783.4911 621.4502	6'''-O-acetylgypenoside XVII isomer	$C_{50}H_{84}O_{19}$	988.5607	L15、N5	-0.20
10.97	829.413	829.5156[M+FA-H]$^-$ 783.4889[M-H]$^-$ 637.4366[M-H-Rha]$^-$ 475.3769[M-H-Rha-Glc]$^-$	Rg_2 isomer	$C_{42}H_{72}O_{13}$	784.4973	L5、L15、Y5、N5	0.77
10.97	793.4395	793.4411[M-H]$^-$ 731.4369 631.3752[M-H-Glc]$^-$ 613.3723[M-H-Glc-H_2O]$^-$ 455.3515[M-H-Glc-GlcA]	CS-Iva isomer	$C_{42}H_{66}O_{14}$	794.4453	L5、L15、Y5、N5	-2.52
11.04	1209.6294	1077.5893[M-H-Xyl]$^-$ 945.5445[M-H-Xyl-Ara(p)]$^-$ 783.4912[M-H-Xyl-Ara(p)-Glc]$^-$	G-Ra_1	$C_{58}H_{98}O_{26}$	1210.6346	L5、L15、Y5、N5	-2.15
11.06	1123.5928	1123.5928[M+HCOO]$^-$ 1077.5920[M-H]$^-$ 945.5500[M-H-Ara(f)]$^-$ 783.4880[M-H-Ara(f)-Glc]$^-$	G-Rc	$C_{53}H_{90}O_{22}$	1078.5924	L5、L15、Y5、N5	-6.87
11.12	683.4397	683.4425[M+HCOO]$^-$ 637.4366[M-H]$^-$ 475.3809[M-H-Glc]$^-$	20(S)-G-Rh_1	$C_{36}H_{62}O_9$	638.4393	L5、L15、Y5、N5	-8.00
11.12	683.4397	683.4425[M+HCOO]$^-$ 637.4366[M-H]$^-$ 475.3809[M-H-Glc]$^-$	20(R)-G-Rh_1	$C_{36}H_{62}O_9$	638.4393	L5、L15、Y5、N5	-8.00
11.19	845.4871	845.4671[M+FA-H]$^-$ 799.4874[M-H]$^-$ 637.4502[M-H-Glc]$^-$ 475.3769[M-H-2Glc]$^-$	Rg_1 isomer	$C_{42}H_{72}O_{14}$	800.4922	L5、L15、Y5、N5	-3.75

11.43	913.518	913.5177[M+FA-H]⁻ 867.5096[M-H]⁻ 799.4952[M-H-C_4H_4O]⁻ 781.4744[M-H-C_4H_4O-H_2O]⁻ 637.4505[M-H-C_4H_4O-Glc]⁻ 619.4416[M-H-C_4H_4O-Glc-H_2O]⁻ 475.3893[M-H-C_4H_4O-2Glc]⁻	koryoginsenoside-R_1/isomer	$C_{46}H_{76}O_{15}$	868.5184	L5、L15、Y5、N5	1.15
11.55	1077.5876	1077.5888[M-H]⁻ 945.5490[M-H-Ara(p)]⁻ 783.4941[M-H-Ara(p)-Glc]⁻	G-Rb_2	$C_{53}H_{90}O_{22}$	1078.5924	L5、L15、Y5、N5	-2.78
11.55	1077.5876	1077.5888[M-H]⁻	G-Rb_3	$C_{53}H_{90}O_{22}$	1078.5924	L5、L15、Y5、N5	-2.78
11.58	825.4972	825.4998[M-H]⁻ 783.4838[M-H-Ac]⁻ 765.4795[M-H-Ac-H_2O]⁻ 621.4407[M-H-Ac-Glc]⁻ 603.4303[M-H-Ac-Glc-H_2O]⁻ 459.3765[M-H-Ac-2Glc]⁻	Rs_3 isomer	$C_{44}H_{74}O_{14}$	826.5079	L5、L15、Y5、N5	3.51
11.6	845.4915	845.4839[M+FA-H]⁻ 799.4891[M-H]⁻ 637.4364[M-H-Glc]⁻ 475.3769[M-H-2Glc]⁻	Rg_1 isomer	$C_{42}H_{72}O_{14}$	800.4922	L5、L15、Y5	-5.88
11.77	841.4951	841.4975[M-H]⁻ 799.4912[M-H-Ac]⁻ 679.4430[M-H-Glc]⁻ 781.4742[M-H-Ac-H_2O]⁻ 637.4256[M-H-Ac-Glc]⁻ 619.4249[M-H-Ac-Glc-H_2O]⁻ 475.3691[M-H-Ac-2Glc]⁻	noto-Rt/isomer	$C_{44}H_{74}O_{15}$	842.5028	L5、L15、Y5、N5	-0.12
11.82	1209.6284	1077.5880[M-H-Xyl]⁻ 1047.5737[M-H-Glc]⁻ 945.5471[M-H-Xyl-Ara(p)]⁻ 783.4912[M-H-Xyl-Ara(f)-Glc]⁻ 765.4903[M-H-Xyl-Ara(f)-Glc-H_2O]⁻ 621.4420[M-H-Xyl-Ara(f)-2Glc]⁻ 459.3802[M-H-Xyl-Ara(f)-3Glc]⁻	G-Ra_1 isomer	$C_{58}H_{98}O_{26}$	1210.6346	L5、L15、Y5、N5	-1.32
11.91	825.4982	825.4972[M-H] - 783.5059[M-H-Ac]⁻ 765.4744[[M-H-Ac-H_2O]⁻ 621.4430[M-H-Ac-Glc]⁻ 475.3766[M-H-Ac-Glc-Rha]⁻	β-D-Glucopyranoside, (3β, 6α,12β,20*R*)-3,12,20-trihydroxydammar-24-en-6-yl 2-*O*-(6-deoxy-α-L-mannopyranosyl)-, 6-acetate isomer	$C_{44}H_{74}O_{14}$	826.5079	L5、L15、Y5、N5	2.30

11.97	969.5422	969.5422 927.5105 783.4861 621.4352 459.3712	6'''-O-acetylgypenoside XVII isomer	$C_{50}H_{84}O_{19}$	988.5607	L5、L15、N5	0.10
12	785.467	785.4922[M+FA-H]⁻ 739.4622[M-H]⁻ 607.4191[M-H-Xyl/Ara]⁻ 475.3762[M-H-2Xyl/Ara]⁻	β-D-Xylopyranoside, (3β, 6α,12β)-3,12,20-trihydroxydammar-24-en-6-yl 2-*O*-Xylopyranosyl-isomer	$C_{40}H_{68}O_{12}$	740.4711	L15、Y5	1.49
12.07	1149.6077	1149.6140[M-H]⁻ 1107.5995[M-H-Ac]⁻ 945.5271[M-H-Ac-Glc]⁻ 783.4751[M-H-Ac-2Glc]⁻	Q-R_1	$C_{56}H_{94}O_{24}$	1150.6135	L5、L15、Y5、N5	-1.74
12.08	869.4886	869.4886[M-H]⁻ 825.5047[M-H-CO_2]⁻ 783.4911[M-H-CO_2-Ac]⁻ 765.4845[M-H-CO_2-Ac-H_2O]⁻ 621.4388[M-H-CO_2-Ac-Glc]⁻ 459.3842[M-H-CO_2-Ac-2Glc]⁻	β-D-Glucopyranoside, (3β, 12β)-20-[[6-*O*-(2-carboxyacetyl)-β-D-glucopyranosyl]oxy]-12-hydroxydammar-24-en-3-yl isomer	$C_{45}H_{74}O_{16}$	870.4977	L5、L15、Y5、N5	1.50
12.1	683.4369	683.4372[M+FA-H]⁻ 637.4343[M-H]⁻ 475.3808[M-H-Glc]⁻	F_1 isomer	$C_{36}H_{62}O_9$	638.4394	L5、L15、Y5、N5	-4.24
12.14	1047.5746	1047.5763[M-H]⁻ 915.5345[M-H-Xyl/Ara]⁻ 783.4937[M-H-2Xyl/Ara]⁻ 621.4351[M-H-2Xyl/Ara-Glc]⁻	Notoginsenoside O isomer	$C_{52}H_{88}O_{21}$	1048.5818	L5、L15、Y5、N5	-0.57
12.17	1013.5757	1013.5757[M-H]⁻ 945.5216[M-H-C_4H_4O]⁻ 783.4902[M-H-C_4H_4O-Glc]⁻ 621.4246[M-H-C_4H_4O-Glc-Glc]⁻ 459.3778[M-H-C_4H_4O-Glc-Glc-Glc]⁻	Quinquenoside I isomer	$C_{52}H_{86}O_{19}$	1014.5763	L5、Y5、N5	-7.10
12.3	829.495	829.5220[M+FA-H]⁻ 783.4936[M-H]⁻ 621.4401[M-H-Glc]⁻ 475.3775[M-H-2Glc]⁻	Rg_2 isomer	$C_{42}H_{72}O_{13}$	784.4973	L5、L15、Y5、N5	-5.23

12.31	825.5011	825.4991[M-H]$^-$ 783.4947[M-H-Ac]$^-$ 765.4786[M-H-Ac-H_2O]$^-$ 663.4434[M-H-Glc]$^-$ 621.4706[M-H-Ac-Glc]$^-$ 459.3755[M-H-Ac-2Glc]$^-$	Rs_3 isomer	$C_{44}H_{74}O_{14}$	826.5079	L5、L15、Y5、N5	-1.21
12.36	763.4303	763.4304[M-H-Xyl/Ara] 631.3865[M-H-Xyl/Ara]$^-$ 455.3466[M-H-Xyl/Ara-GlcA]$^-$	3-O-[R-L-Arabinopyranosyl(1f2)-β-D-glucuronopyranosyl] oleanolic acid isomer	$C_{41}H_{64}O_{13}$	764.4347	L5、L15、Y5、N5	-4.45
12.4	929.5482	929.5522[M-H]$^-$ 767.5045[M-H-Glc]$^-$ 605.4526[M-H-2Glc]$^-$ 459.3850[M-H-2Glc-Rha]$^-$	Gypenoside X isomer	$C_{48}H_{82}O_{17}$	930.5552	L5、L15、Y5、N5	-0.86
12.45	1119.5937	1119.6016[M-H]$^-$ 1077.5889[M-H-Ac]$^-$ 1059.5754[M-H-Ac-H_2O]$^-$ 945.5460[M-H-Ac-Xyl/Ara]$^-$ 927.5327[M-H-Ac-Xyl/Ara-H_2O]$^-$ 915.5334[M-H-Ac-Glc]$^-$ 783.4920[M-H-Ac-Glc-Xyl/Ara]$^-$ 765.4832[M-H-Ac-Glc-Xyl/Ara-H_2O]$^-$ 621.4377[M-H-Ac-2Glc-Xyl/Ara]$^-$ 459.3698[M-H-Ac-3Glc-Xyl/Ara]$^-$	Rs_1 isomer	$C_{55}H_{92}O_{23}$	1120.6029	L5、L15、Y5、N5	1.25
12.56	991.5505	991.5525[M+HCOO]$^-$ 945.5460[M-H]$^-$ 783.4973[M-H-Glc]$^-$ 621.4388[M-H-2Glc]$^-$	G-Rd	$C_{48}H_{82}O_{18}$	946.5501	L5、L15、Y5、N5	-3.91
12.68	1077.5874	1077.5859[M-H]$^-$ 945.5425[M-H-Xyl/Ara]$^-$ 783.4687[M-H-Xyl/Ara-Glc]$^-$ 621.4494[M-H-Xyl/Ara-2Glc]$^-$ 459.3653[M-H-Xyl/Ara-3Glc]$^-$	Rb_1 isomer	$C_{53}H_{90}O_{21}$	1078.5924	L15、Y5、N5	-2.60
12.76	765.4793	765.4793[M-H]$^-$ 603.3942[M-H-Glc]$^-$ 441.3382[M-H-2Glc]$^-$	G-Rk_1	$C_{42}H_{70}O_{12}$	766.4867	L5、L15、Y5、N5	-0.52
12.79	1149.6056	1149.6084[M-H]$^-$ 1107.5950[M-H-Ac]$^-$ 1089.5862[M-H-Ac-H_2O]$^-$ 945.5474[M-H-Ac-Glc]$^-$ 783.4985[M-H-Ac-2Glc]$^-$ 621.4451[M-H-Ac-3Glc]$^-$ 459.3763[M-H-Ac-4Glc]$^-$	Q-R_1 isomer	$C_{56}H_{94}O_{24}$	1150.6135	L5、L15、Y5、N5	0.09

12.9	1119.5972	1119.6023[M-H]⁻ 1077.5944[M-H-C_2H_2O]⁻ 1077.6355[M-H-C_2H_2O]⁻ 1059.5786[M-H-Ac]⁻	Rs_2	$C_{55}H_{92}O_{23}$	1120.6029	L5、L15、Y5、N5	-1.88
12.9	1119.5972	1119.6023[M-H]⁻ 1077.5944[M-H-C_2H_2O]⁻ 1077.6355[M-H-C_2H_2O]⁻ 1059.5786[M-H-Ac]⁻	Rs_1	$C_{55}H_{92}O_{23}$	1120.6029	L5、L15、Y5、N5	-1.88
12.99	871.5036	871.5212[M+FA-H]⁻ 825.5077[M-H]⁻ 783.4969[M-H-Ac]⁻ 637.4444[M-H-Ac-Rha]⁻ 475.3821[M-H-Ac-Rha-Glc]⁻	β-D-Glucopyranoside, (3β, 6α,12β,20*R*)-3,12,20-trihydroxydammar-24-en-6-yl 2-*O*-(6-deoxy-α-L-mannopyranosyl)-, 6-acetate isomer	$C_{44}H_{74}O_{14}$	826.5079	L5、L15、Y5、N5	-9.21
13	1047.5722	1047.5785[M-H]⁻ 915.5464[M-H-Xyl/Ara]⁻ 783.4876[M-H-2Xyl/Ara]⁻ 621.4268[M-H-2Xyl/Ara-Glc]⁻ 459.4064[M-H-2Xyl/Ara-2Glc]⁻	Notoginsenoside O isomer	$C_{52}H_{88}O_{21}$	1048.5818	L5、L15、Y5、N5	1.72
13.05	1175.6198	1175.6281[M-H]⁻ 1107.6023[M-H-C_4H_4O]⁻ 1089.5882[M-H-C_4H_4O-H_2O]⁻ 945.5458[M-H-C_4H_4O-Glc]⁻ 783.4935[M-H-C_4H_4O-2Glc]⁻ 765.4780[M-H-C_4H_4O-2Glc-H_2O]⁻ 621.4361[M-H-C_4H_4O-3Glc]⁻ 459.3930[M-H-C_4H_4O-4Glc]⁻	β-D-Glucopyranoside, (3β, 12β)-20-[(6-*O*-β-D-glucopyranosyl-β-D-glucopyranosyl)oxy]-12-hydroxydammar-24-en-3-yl 2-*O*-[6-*O*-[(2*E*)-1-oxo-2-buten-1-yl]-β-D-glucopyranosyl]-isomer	$C_{58}H_{96}O_{24}$	1176.6292	L5、L15、Y5、N5	1.36
13.18	945.5438	945.5491[M-H]⁻ 783.4911[M-H-Glc]⁻ 621.4233[M-H-2Glc]⁻ 459.3912[M-H-3Glc]⁻	Rd isomer	$C_{48}H_{82}O_{18}$	946.5501	L5、L15、Y5、N5	-1.59
13.19	913.5175	913.5067[M+FA-H]⁻ 867.5118[M-H]⁻ 799.4870[M-H-C_4H_4O]⁻ 637.4354[M-H-C_4H_4O-Glc]⁻ 475.3766[M-H-C_4H_4O-2Glc]⁻	koryoginsenoside-R_1/isomer	$C_{46}H_{76}O_{15}$	868.5184	L5、L15、Y5、N5	-1.38

13.42	1145.6106	1145.6186[M-H]⁻ 1077.5918[M-H-C_4H_4O]⁻ 1059.5801[M-H-C_4H_4O-H_2O]⁻ 945.5450[M-H-C_4H_4O-Xyl/Ara]⁻ 783.4872[M-H-C4H4O-Xyl/Ara-Glc]⁻ 765.4993[M-H-C_4H_4O-Xyl/Ara-Glc-H_2O]⁻ 621.4356[M-H-C_4H_4O-Xyl/Ara-2Glc]⁻ 459.3738[M-H-C_4H_4O-Xyl/Ara-3Glc]⁻	ginsenoside Ra$_7$ isomer	$C_{57}H_{94}O_{23}$	1146.6186	L5、L15、Y5、N5	0.17
13.45	1119.5962	1119.5975[M-H]⁻ 1077.5926[M-H-Ac]⁻ 1059.5864[M-H-Ac-H_2O]⁻ 945.5513[M-H-Ac-Xyl/Ara]⁻ 915.5329[M-H-Ac-Glc]⁻ 783.5016[M-H-Ac-Glc-Xyl/Ara]⁻ 621.4504[M-H-Ac-2Glc-Xyl/Ara]⁻ 459.5302[M-H-Ac-3Glc-Xyl/Ara]⁻	Rs$_1$ isomer	$C_{55}H_{92}O_{23}$	1120.6029	L5、L15、Y5、N5	-0.98
13.49	969.5092	969.516[M-H]⁻ 807.4603[M-H-Glc]⁻ 609.3828[M-H-2Glc-2H2O]⁻ 455.3540[M-H-2Glc-gluA-CH2]⁻	G-Ro methyl ester	$C_{48}H_{76}O_{19}$	970.5137	L5、L15、Y5、N5	-3.40
13.57	827.4787	827.4883[M+FA-H]⁻ 781.4707[M-H]⁻ 619.4312[M-H-Glc]⁻ 457.3872[M-H-2Glc]⁻	G-Rh$_{15}$/isomer	$C_{42}H_{70}O_{13}$	782.4816	L5、L15、Y5、N5	3.97
13.77	827.4814	827.4802[M+HCOO]⁻ 781.4798[M-H]⁻ 619.4225[M-H-Glc]⁻ 457.3717[M-H-2Glc]⁻	G-Rg$_9$	$C_{42}H_{70}O_{13}$	782.4816	L5、L15、Y5、N5	-7.60
13.78	987.5549	987.5549[M-H]⁻ 945.5439[M-H-Ac]⁻ 927.5337[M-H-Ac-H_2O]⁻ 783.4908[M-H-Ac-Glc]⁻ 765.4852[M-H-Ac-H_2O-Glc]⁻ 621.4333[M-H-Ac-2Glc]⁻ 459.4046[M-H-Ac-3Glc]⁻	6'''-O-acetylgypenoside XVII isomer	$C_{50}H_{84}O_{19}$	988.5607	L5、L15、Y5、N5	-2.03
13.78	915.5343	915.5353[M-H]⁻ 783.4908[M-H-Ara/Xyl]⁻ 621.4370[M-H-Ara/Xyl-Glc]⁻ 459.3812[M-H-Ara/Xyl-2Glc]⁻	CS-III isomer	$C_{47}H_{80}O_{17}$	916.5396	L5、L15、Y5、N5	-2.73
13.81	913.5163	913.5406[M+FA-H]⁻ 867.5107[M-H]⁻ 799.4865[M-H-C_4H_4O]⁻ 781.4857[M-H-C_4H_4O-H_2O]⁻ 637.4456[M-H-C_4H_4O-Glc]⁻ 571.4121 475.3700[M-H-C_4H_4O-2Glc]	koryoginsenoside-R$_1$/isomer	$C_{46}H_{76}O_{15}$	868.5184	L5、N5	-0.12
13.89	637.4332	637.4349[M-H]⁻ 475.3828[M-H-Glc]⁻	G-Rh$_{19}$	$C_{36}H_{62}O_9$	638.4394	L5、L15、Y5、N5	-2.51

14.08	807.4911	807.4943[M-H]⁻ 765.4824[M-H-Ac]⁻ 747.4726[M-H-Ac-H_2O]⁻ 603.4296[M-H-Ac-Glc]⁻ 441.3694[M-H-Ac-2Glc]⁻	Rs_4 isomer	$C_{44}H_{72}O_{13}$	808.4973	L5、L15、Y5、N5	-1.98
14.1	783.4914	783.4913[M-H]⁻ 621.4361[M-H-Glc]⁻	DHDGG	$C_{42}H_{72}O_{13}$	784.4973	L5、L15	-2.43
14.26	987.5523	987.5542[M-H]⁻ 945.5451[M-H-Ac]⁻ 927.5273[M-H-Ac-H_2O]⁻ 783.4858[M-H-Ac-Glc]⁻ 765.4884[M-H-Ac-Glc-H_2O]⁻ 621.4309[M-H-Ac-Glc-Glc]⁻ 459.3818[M-H-Ac-Glc-Glc-Glc]	6'''-O-acetylgypenoside XVII isomer	$C_{50}H_{84}O_{19}$	988.5607	L5、L15、Y5、N5	0.61
14.39	811.4871	811.4896[M+HCOO]⁻ 765.4833[M-H]⁻ 619.4246[M-H-Rha]⁻ 457.3736[M-H-Rha-Glc]⁻	G-Rg_6	$C_{42}H_{70}O_{12}$	766.4867	L5、L15、Y5、N5	-5.75
14.39	765.4814	765.482[M-H]⁻ 619.4232[M-H-Rha]⁻	G-F_4	$C_{42}H_{70}O_{12}$	766.4867	L5、L15、Y5、N5	-3.27
14.46	797.4706	751.4951[M-H]⁻ 797.4733[M+HCOO]⁻ 619.4275[M-H-Xyl]⁻	DHDXG	$C_{42}H_{72}O_{11}$	752.5075	L5、L15、Y5、N5	6.12
14.5	807.4544	645.4015[M-H-Glc]⁻ 807.4582[M-H]⁻	CS-Iva methyl ester	$C_{43}H_{68}O_{14}$	808.4609	L5、L15、Y5	-1.61
14.5	725.4486	725.4466[M+FA-H]⁻ 679.4402[M-H]⁻ 637.4289[M-H-Ac]⁻ 475.3965[M-H-Ac-Glc]⁻	β-D-Glucopyranoside, (3β, 6α,12β,20S)-6-(acetyloxy) -3,12-dihydroxydammar-24-en-20-yl isomer	$C_{38}H_{64}O_{10}$	680.4499	L5、L15、Y5、N5	2.80
14.68	1059.577	1059.5753[M+FA-H]⁻ 1013.5717[M-H]⁻ 945.5444[M-H-C_4H_4O]⁻ 927.5337[M-H-C_4H_4O-H_2O]⁻ 783.4901[M-H-C_4H_4O-Glc]⁻ 765.4861[M-H-C_4H_4O-Glc-H_2O]⁻ 621.4401[M-H-C_4H_4O-2Glc]⁻ 459.3767[M-H-C_4H_4O-3Glc]⁻	Quinquenoside I isomer	$C_{52}H_{86}O_{19}$	1014.5763	L5、L15、Y5、N5	-3.16
14.68	975.5552	975.5552[M+FA-H]⁻ 929.5511[M-H]⁻ 783.4878[M-H-Rha]⁻ 621.4535[M-H-Rha-Glc]⁻ 459.3782[M-H-Rha-Glc-Glc]	Gypenoside X isomer	$C_{48}H_{82}O_{17}$	930.5552	L5、L15、Y5、N5	-3.98
14.68	811.4877	811.4762[M+FA-H]⁻ 765.4814[M-H]⁻ 619.4101[M-H-Rha]⁻ 457.3726[M-H-Rha-Glc]⁻	G-Rg_6 isomer	$C_{42}H_{70}O_{12}$	766.4867	L5、L15、Y5、N5	-3.27

14.83	665.4286	665.4299[M+HCOO]⁻ 619.4241[M-H]⁻	G-Rk_3	$C_{36}H_{60}O_8$	620.4288	L5、L15、Y5、N5	-5.00
14.83	665.4286	665.4299[M+HCOO]⁻ 619.4241[M-H]⁻	G-Rh_4	$C_{36}H_{60}O_8$	620.4288	L5、L15、Y5、N5	-5.00
14.98	783.491	783.4913[M-H]⁻ 621.4361[M-H-Glc]⁻	G-F_2	$C_{42}H_{72}O_{13}$	784.4973	L5、L15、Y5、N5	-1.91
15.34	827.4784	827.4749[M+FA-H]⁻ 781.4764[M-H]⁻ 619.4216[M-H-Glc]⁻ 457.3858[M-H-2Glc]⁻	G-Rh_{15}/isomer	$C_{42}H_{70}O_{13}$	782.4816	L5、L15、Y5、N5	-3.33
15.39	1013.5701	1013.5723[M-H]⁻ 945.5463[M-H-C_4H_4O]⁻ 927.5273[M-H-C_4H_4O-H_2O]⁻ 783.4935[M-H-C_4H_4O-Glc]⁻ 765.4950[M-H-C_4H_4O-Glc-H_2O]⁻ 621.4445[M-H-C_4H_4O-2Glc]⁻ 459.3787[M-H-C_4H_4O-3Glc]⁻	Quinquenoside I isomer	$C_{52}H_{86}O_{19}$	1014.5763	L5、L15、Y5、N5	-1.58
15.6	1029.5647	1029.5679[M-H]⁻ 987.5458[M-H-Ac]⁻ 945.5502[M-H-2Ac]⁻ 783.5046[M-H-2Ac-Glc]⁻ 621.4294[M-H-2Ac-2Glc]⁻ 459.3784[M-H-2Ac-3Glc]⁻	Diacetyl-G-Rd isomer	$C_{52}H_{86}O_{20}$	1030.5712	L5、L15、Y5、N5	-1.26
16.16	851.51	851.5100[M-H]⁻ 783.4875[M-H-C_4H_4O]⁻ 621.4525[M-H-C_4H_4O-Glc]⁻ 459.3966[M-H-C_4H_4O-2Glc]⁻	β-D-Glucopyranoside, (3β, 12β)-3-(β-D-glucopyranosyloxy)-12-hydroxydammar-24-en-20-yl, 6-(2*E*)-2-butenoate isomer	$C_{46}H_{76}O_{14}$	852.5235	L15、Y5	6.69
16.18	783.4925	783.4947[M-H]⁻ 621.4389[M-H-Glc]⁻ 459.3840[M-H-2Glc]⁻	20(S)-G-Rg_3	$C_{42}H_{72}O_{13}$	784.4973	L5、L15、Y5、N5	-3.83
16.18	783.4925	783.4947[M-H]⁻ 621.4389[M-H-Glc]⁻ 459.3840[M-H-2Glc]⁻	20(R)-G-Rg_3	$C_{42}H_{72}O_{13}$	784.4973	L5、L15、Y5、N5	-3.83

16.46	829.4954	829.5001[M+FA-H]$^-$ 783.4931[M-H]$^-$ 621.4475[M-H-Glc]$^-$ 459.3833[M-H-2Glc]$^-$	F_2 isomer	$C_{42}H_{72}O_{13}$	784.4973	L5、L15、Y5、N5	-4.59
16.97	459.3845	459.3845[M+H]+ 441.3741[M+H-H_2O]+	DEDT	$C_{30}H_{50}O_3$	458.376	L5、L15、Y5、N5	-1.52
17.01	475.3787	475.3786[M-H]$^-$ 391.2836[M-H-C_6H_{12}]$^-$	20(S)-PPT	$C_{30}H_{52}O_4$	476.3866	L5、L15、Y5、N5	0.21
17.01	475.3787	475.3786[M-H]$^-$ 391.2836[M-H-C_6H_{12}]$^-$	20(R)-PPT	$C_{30}H_{52}O_4$	476.3866	L5、L15、Y5、N5	0.21
17.18	799.4847	799.4891[M+FA-H]$^-$ 753.4784[M-H]$^-$ 621.4433[M-H-Xyl/Ara]$^-$ 459.3814[M-H-Xyl/Ara-Glc]$^-$	G-C-Y isomer	$C_{41}H_{70}O_{12}$	754.4867	L5、L15、Y5、N5	0.66
17.35	827.4803	827.4759[M+FA-H]$^-$ 781.4773[M-H]$^-$ 619.4345[M-H-Glc]$^-$ 457.3735[M-H-2Glc]$^-$	G-Rh_{15}/isomer	$C_{42}H_{70}O_{13}$	782.4816	L5、L15、Y5、N5	-4.48
17.55	799.4844	799.4859[M+FA-H]$^-$ 753.4824[M-H]$^-$ 621.4417[M-H-Xyl/Ara]$^-$ 459.3995[M-H-Xyl/Ara-Glc]$^-$	G-C-Y isomer	$C_{41}H_{70}O_{12}$	754.4867	L5、Y5、N5	-4.65
17.7	871.5045	871.5041[M+FA-H]$^-$ 825.4979[M-H]$^-$ 783.4866[M-H-Ac]$^-$ 621.4428[M-H-Ac-Glc]$^-$ 459.3854[M-H-Ac-Glc-Glc]	Rs_3 isomer	$C_{44}H_{74}O_{14}$	826.5079	L5、Y5、N5	2.67
18.33	871.5073	871.4739[M+HCOO]$^-$ 825.5006[M-H]$^-$ 783.4949[M-H-C_2H_2O] 765.4761[M-H-AC]$^-$ 621.4482[M-H-Glc]$^-$ 459.3823[M-H-2Glc]$^-$	20(S)-G-Rs_3	$C_{44}H_{74}O_{14}$	826.5079	L5、L15、Y5、N5	-0.61
18.33	871.5073	871.4739[M+HCOO]$^-$ 825.5006[M-H]$^-$ 783.4949[M-H-C_2H_2O] 765.4761[M-H-Ac]$^-$ 621.4482[M-H-Glc]$^-$ 459.3823[M-H-2Glc]$^-$	20(R)-G-Rs_3	$C_{44}H_{74}O_{14}$	826.5079	L5、L15、Y5、N5	-0.61
18.44	807.4548	807.4572[M-H]$^-$ 609.3828[M-H-Glc-2H_2O]$^-$ 537.3592 455.3544[M-H-GlcA-6'-O-Me-Glc]	ZB-RI-6‘ methyl ester	$C_{43}H_{68}O_{14}$	808.4609	L15	-2.11
18.44	807.4889	807.4889[M-H]$^-$ 609.3828[M-H-Glc-2H_2O]$^-$	Rs_4	$C_{44}H_{72}O_{13}$	808.4973	L15	0.74

18.45	797.4658	797.4840[M+FA-H]$^-$ 751.4632[M-H]$^-$ 619.4250[M-H-Xyl/Ara]$^-$ 457.3681[M-H-Xyl/Ara-Glc]$^-$	noto-T_5 isomer	$C_{41}H_{68}O_{12}$	752.4711	L5、L15、Y5、N5	0.13
19.06	663.4096	663.4111[M+FA-H]$^-$ 617.4134[M-H]$^-$ 455.3506[M-H-Glc]$^-$	Oleanolic acid-28-O-β-D-glucopyranoside/isomer	$C_{36}H_{58}O_8$	618.4132	L5、L15、Y5、N5	-12.96
19.07	665.4161	665.4419[M+FA-H]$^-$ 619.4148[M-H]$^-$ 457.3730[M-H-Glc]$^-$	G-Rh_4 isomer	$C_{36}H_{60}O_8$	620.4288	N5	10.00
19.9	667.4459	621.4392[M-H]$^-$ 459.3852[M-H-Glc]$^-$	G-C-K	$C_{36}H_{62}O_8$	622.4445	L5、L15、Y5、N5	-4.02
19.93	765.4815	765.4820[M-H]$^-$ 603.4280[M-H-Glc]$^-$ 441.3389[M-H-2Glc]$^-$	G-Rg_5	$C_{42}H_{70}O_{12}$	766.4867	L5、L15、Y5、N5	-3.40
20.06	667.4412	667.4391[M+FA-H]$^-$ 621.4330[M-H]$^-$ 459.3945[M-H-Glc]$^-$	G-C-K isomer	$C_{36}H_{62}O_8$	622.4445	L5、L15、Y5	5.95
20.68	649.346	649.3596[M+FA-H]$^-$ 603.4193[M-H]$^-$ 441.2927[M-H-Glc]$^-$	Rh_3 isomer	$C_{36}H_{60}O_7$	604.4339	L5、L15、Y5、N5	0.33
21.02	621.4371	621.4382[M-H]$^-$ 459.3831[M-H-Glc]$^-$	20(S)-G-Rh_2	$C_{36}H_{62}O_8$	622.4445	L5、L15、Y5、N5	-0.64
21.02	621.4371	621.4382[M-H]$^-$ 459.3831[M-H-Glc]$^-$	20(S)-G-Rh_2	$C_{36}H_{62}O_8$	622.4445	L5、L15、Y5、N5	-0.64
21.29	665.4263	665.4260[M+FA-H]$^-$ 619.4224[M-H]$^-$ 457.3760[M-H-Glc]$^-$	G-Rh_4 isomer	$C_{36}H_{60}O_8$	620.4288	L5、L15、Y5、N5	-2.26
21.68	459.3819	459.3819[M+H]+ 441.3741[M+H-H20]+	DDT	$C_{30}H_{50}O_3$	458.376	L5、L15、Y5、N5	4.14
21.91	807.4905	807.4898[M-H]$^-$ 765.4821[M-H-Ac] 747.4694[M-H-Ac-H_2O]$^-$ 603.4212[M-H-Ac-Glc]$^-$ 441.3721[M-H-Ac-2Glc]	Rs4 isomer	$C_{44}H_{72}O_{13}$	808.4973	L5、L15、Y5	-1.24
23.72	649.4337	649.4332[M+HCOO]$^-$ 603.4311[M-H]$^-$	G-Rh_3	$C_{36}H_{60}O_7$	604.4339	L5、L15、Y5、N5	-8.29

23.72	649.4337	649.4332[M+HCOO]$^-$ 603.4311[M-H]$^-$	G-Rk$_2$	$C_{36}H_{60}O_7$	604.4339	L5、L15、Y5、N5	-8.29
25.54	505.3891	505.3847[M+HCOO]$^-$ 459.3830[M-H]$^-$	20(S)-PPD	$C_{30}H_{52}O_3$	460.3916	L5、L15、Y5、N5	1.74
25.54	505.3891	505.3847[M+HCOO]$^-$ 459.3830[M-H]$^-$	20(S)-PPD	$C_{30}H_{52}O_3$	460.3916	L5、L15、Y5、N5	1.74

Table S2 MS and MS/MS of the 75 potential new ginsenosides

No	RT	Found Mass	MS/MS	Identification	Name	Formula	Theoretical Mass (Da)	Sources	Error (ppm)
1	1.39	973.5384	973.5392[M-H]$^-$ 931.5222[M-H-Ac]$^-$ 913.5125[M-H-Ac-H_2O]$^-$ 799.4752[M-H-Ac-Xyl/Ara]$^-$ 637.4390[M-H-Ac-Xyl/Ara-Glc]$^-$ 475.3570[M-H-Ac-Xyl/Ara-2Glc]$^-$	PPT-Glc-Glc-Xyl/Ara-acetyl	acetyl-NG-R1A	$C_{49}H_{82}O_{19}$	974.545	L5,L15,Y5	-1.23
2	1.4	1017.527	1017.5282[M-H]$^-$ 973.5391[M-H-CO_2]$^-$ 931.5300[M-H-CO_2-Ac]$^-$ 799.4940[M-H-CO_2-Ac-Xyl/Ara]$^-$ 637.4293[M-H-CO_2-Ac-Xyl/Ara-Glc]$^-$ 475.3634[M-H-CO_2-Ac-Xyl/Ara-2Glc]$^-$	PPT-Glc-Glc-Xyl/Ara-malonyl	malonyl-NG-R1	$C_{50}H_{82}O_{21}$	1018.5349	L5,L15,Y5,N5	0.10
3	1.48	739.4243	739.3379[M-H]$^-$ 695.4553[M-H-CO_2]$^-$ 653.4397[M-H-CO_2-Ac]$^-$ 635.4165[M-H-CO_2-Ac-H_2O]$^-$ 491.3845[M-H-CO_2-Ac-Glc]$^-$	PG-Glc-malonyl	malonyl-PG-RT5	$C_{39}H_{64}O_{13}$	740.4347	N5	3.52
4	1.5	961.5377	961.5370[M-H]$^-$ 799.4818[M-H-Glc]$^-$ 781.4803[M-H-Glc-H_2O]$^-$ 653.4289[M-H-Glc-Rha]$^-$ 491.3668[M-H-2Glc-Rha]	PG-Glc-Glc-Rha	PG-F11-G1	$C_{48}H_{82}O_{19}$	962.545	L5,L15,Y5,N5	-0.52
5	1.54	913.5142	913.5169[M-H]$^-$ 781.4893[M-H-Xyl/Ara]$^-$ 619.4175[M-H-Xyl/Ara-Glc]$^-$ 457.3573[M-H-Xyl/Ara-2Glc]$^-$	PPT-C17-V1/V2-Glc-Glc-Xyl/Ara	DHDXG-G1	$C_{47}H_{78}O_{17}$	914.5239	L15	2.08

6	1.69	1047.5372	1047.4597[M-H]$^-$ 1003.5481[M-H-CO_2]$^-$ 961.5407[M-H-CO_2-Ac]$^-$ 943.5309[M-H-CO_2-Ac-H_2O]$^-$ 799.4768[M-H-CO_2-Ac-Glc]$^-$ 637.4257[M-H-CO_2-Ac-Glc-Glc]$^-$ 475.3680[M-H-CO_2-Ac-3Glc]	PPT-Glc-Glc-Glc-malonyl	malonyl-Re2-A	$C_{51}H_{84}O_{22}$	1048.5454	L5,Y5,N5	0.38
7	1.9	927.5	927.5353[M-H]$^-$ 883.4862[M-H-CO_2]$^-$ 841.4944[M-H-CO_2-Ac]$^-$ 799.5072[M-H-CO_2-2Ac]$^-$ 781.4763[M-H-CO_2-2Ac-H_2O]$^-$ 637.4274[M-H-CO_2-2Ac-Glc]$^-$ 475.3788[M-H-CO_2-2Ac-2Glc]$^-$	PPT-Glc-Glc-malonyl-acetyl	malonyl-acetyl-Rg1A	$C_{47}H_{76}O_{18}$	928.5032	L5,L15,Y5,N5	-4.96
8	1.9	927.5306	927.5353[M-H]$^-$ 799.5072[M-H-Rha+H_2O]$^-$ 781.4763[M-H-Rha]$^-$ 637.4274[M-H-Rha-Glc+H_2O]$^-$ 619.4185[M-H-Rha-Glc]$^-$ 475.3788[M-H-Rha-Glc-Glc+H_2O]$^-$	PPT-C17-V1-Glc-Glc-Rha	Rg6-G1	$C_{48}H_{80}O_{17}$	928.5396	L5,L15,Y5,N5	1.29
9	2.02	953.5194	953.5194[M-H]$^-$ 909.4709[M-H-CO_2]$^-$ 867.4609[M-H-CO_2-Ac]$^-$ 799.4744[M-H-CO_2-Ac-C_4H_4O]$^-$ 637.4358[M-H-CO_2-Ac-C_4H_4O-Glc]$^-$ 475.3771[M-H-CO_2-Ac-C_4H_4O-2Glc]$^-$	PPT-Glc-Glc-malonyl-butene acyl	malonyl-butene acyl-Rg1	$C_{49}H_{78}O_{18}$	954.5188	L5,L15,Y5,N5	-8.81
10	2.36	929.5391	929.5237[M-H]$^-$ 783.4800[M-H-Rha]$^-$ 621.4264[M-H-Rha-Glc]$^-$ 475.4109[M-H-Rha-Glc-Rha]$^-$	PPT-Rha-Glc-Rha	Rg2-G1	$C_{48}H_{82}O_{17}$	930.5552	N5	8.93
11	2.48	961.5352	961.5350[M-H]$^-$ 815.4813[M-H-Rha]$^-$ 653.4181[M-H-Rha-Glc]$^-$ 491.3826[M-H-Rha-Glc-Glc]$^-$	PG-Glc-Glc-Rha	PG-F11-G2	$C_{48}H_{82}O_{19}$	962.545	L5,Y5,N5	2.08
12	3.36	1139.5866	1139.5807[M+FA-H]$^-$ 1093.5745[M-H]$^-$ 961.5355[M-H-Ara/Xyl]$^-$ 815.4787[M-H-Xyl/Ara-Rha]$^-$ 653.4234[M-H-Xyl/Ara-Rha-Glc]$^-$ 491.3770[M-H-Xyl/Ara-Rha-2Glc]$^-$	PG-Glc-Glc-Xyl/Ara-Rha	PG-F11-GX1	$C_{53}H_{90}O_{23}$	1094.5873	L15,Y5,N5	4.57
13	4.68	833.4806	833.4836[M+FA-H]$^-$ 787.4791[M-H]$^-$ 655.4330[M-H-Xyl/Ara]$^-$ 493.3797[M-H-Xyl/Ara-Glc]$^-$	PPT-C17-V3/V4-Glc-Xyl/Ara	Rsyz1	$C_{41}H_{74}O_{14}$	788.4922	L15,Y5	6.73
14	4.81	855.4718	855.3784[M-H]$^-$ 811.4839[M-H-CO_2]$^-$ 679.4563[M-H-CO_2-Xyl/Ara]$^-$ 637.4364[M-H-CO_2-Ac-Xyl/Ara]$^-$ 475.3786[M-H-CO_2-Ac-Xyl/Ara-Glc]$^-$	PPT-Glc-Xyl/Ara-malonyl	malonyl-F3A	$C_{44}H_{72}O_{16}$	856.482	Y5,N5	2.81

15	4.91	927.4958	927.5027[M-H]⁻ 883.5069[M-H-CO_2]⁻ 841.4992[M-H-CO_2-Ac]⁻ 799.4887[M-H-CO_2-2Ac]⁻ 637.4183[M-H-CO_2-2Ac-Glc]⁻ 475.3675[M-H-CO_2-2Ac-2Glc]⁻	PPT-Glc-Glc-malonyl-acetyl	malonyl-acetyl-Rg1B	$C_{47}H_{76}O_{18}$	928.5032	L5,L15,Y5,N5	-0.43
16	4.94	1063.5711	1063.5715[M-H]⁻ 931.5293[M-H-Xyl/Ara]⁻ 913.4992[M-H-Xyl/Ara-H2O]⁻ 799.4959[M-H-2Xyl/Ara]⁻ 637.4197[M-H-2Xyl/Ara-Glc]⁻ 475.3858[M-H-2Xyl/Ara-2Glc]⁻	PPT-Glc-Glc-Xyl/Ara-Xyl/Ara	Re1-X1	$C_{52}H_{88}O_{22}$	1064.5767	L5,L15,Y5,N5	-2.07
17	5.23	867.4752	867.4691[M-H]⁻ 823.4857[M-H-CO_2]⁻ 781.4791[M-H-CO_2-Ac]⁻ 763.4678[M-H-CO_2-Ac-H_2O]⁻ 619.4139[M-H-CO_2-Ac-Glc]⁻ 601.4099[M-H-CO_2-Ac-Glc-H_2O]⁻ 457.3628[M-H-CO_2-Ac-2Glc]⁻	PPT-C17-V1/V2-Glc-Glc-malonyl	malonyl-Rg9	$C_{45}H_{72}O_{16}$	868.482	L5,L15	-1.15
18	5.24	1047.5332	1047.5392[M-H]⁻ 1003.5535[M-H-CO_2]⁻ 961.5421[M-H-CO_2-Ac]⁻ 799.4758[M-H-CO_2-Ac-Glc]⁻ 637.4244[M-H-CO_2-Ac-2Glc]⁻ 475.3817[M-H-CO_2-Ac-3Glc]⁻	PPT-Glc-Glc-Glc-malonyl	malonyl-Re2-B	$C_{51}H_{84}O_{22}$	1048.5454	L15,Y5,N5	4.20
19	5.27	823.4844	823.4831[M-H]⁻ 781.4692[M-H-Ac]⁻ 763.4840[M-H-Ac-H_2O]⁻ 619.4307[M-H-Ac-Glc]⁻ 457.3747[M-H-Ac-2Glc]⁻	PPT-C17-V1/V2-Glc-Glc-acetyl	acetyl-Rg9	$C_{44}H_{72}O_{14}$	824.4922	L15,Y5	0.00
20	5.52	1121.5748	1121.5820[M-H]⁻ 959.5284[M-H-Glc]⁻ 797.4781[M-H-2Glc]⁻ 635.4177[M-H-3Glc] 473.3593[M-H-4Glc]⁻	PPT-C17-V5-Glc-Glc-Glc-Glc	Rh-4G	$C_{54}H_{90}O_{24}$	1122.5822	L5,L15,Y5,N5	-0.36
21	5.62	975.5131	975.5230[M+FA-H]⁻ 929.5099[M-H]⁻ 797.4873[M-H-Xyl/Ara]⁻ 635.4114[M-H-Xyl/Ara-Glc]⁻ 617.4074[M-H-Xyl/Ara-Glc-H_2O]⁻ 473.3614[M-H-Xyl/Ara-2Glc]⁻	PPT-C17-V5-Glc-Glc-Xyl/Ara	RH-2GX	$C_{47}H_{78}O_{18}$	930.5188	L15	1.18
22	5.76	927.4945	927.5334[M-H]⁻ 883.5071[M-H-CO_2]⁻ 841.4904[M-H-CO_2-Ac]⁻ 799.5156[M-H-CO_2-2Ac]⁻ 637.4343[M-H-CO_2-2Ac-Glc]⁻ 475.3852[M-H-CO_2-2Ac-2Glc]⁻	PPT-Glc-Glc-malonyl-acetyl	malonyl-acrtyl-Rg1C	$C_{47}H_{76}O_{18}$	928.5032	L5,L15	0.97
23	5.94	855.4744	811.4870[M-CO_2]⁻ 769.4764[M-H-CO_2-Ac]⁻ 637.4296[M-H-CO_2-Ac-Xyl/Ara]⁻ 475.3806[M-H-CO_2-Ac-Xyl/Ara-Glc]⁻	PPT-Glc-Xyl/Ara-malonyl	malonyl-F3B	$C_{44}H_{72}O_{16}$	856.482	L15,Y5	-0.23

24	6.09	943.5259	943.5243[M-H]$^-$ 797.4652[M-H-Rha]$^-$ 635.4301[M-H-Rha-Glc]$^-$ 473.3596[M-H-Rha-2Glc]$^-$	PPT-C17-V5-Glc-Glc-Rha	Rh-2GR	$C_{48}H_{80}O_{18}$	944.5345	L5,Y5,N5	0.85
25	6.14	1073.5542	1073.5571[M-H]$^-$ 1029.5684[M-H-CO$_2$]$^-$ 987.5569[M-H-CO$_2$-Ac]$^-$ 945.5459[M-H-CO$_2$-2Ac]$^-$ 783.4915[M-H-CO$_2$-2Ac-Glc]$^-$ 621.4386[M-H-CO$_2$-2Ac-2Glc]$^-$ 459.3961[M-H-CO$_2$-2Ac-3Glc]$^-$	PPD-Glc-Glc-Glc-malonyl-acetyl	malonyl-acetyl-Rd	$C_{53}H_{86}O_{22}$	1074.5611	L15,Y5,N5	-0.84
26	6.15	1117.5416	1117.5461[M-H]$^-$ 1073.5553[M-H-CO$_2$]$^-$ 1029.5668[M-H-2CO$_2$]$^-$ 987.5565[M-H-2CO$_2$-Ac]$^-$ 969.5464[M-H-2CO$_2$-Ac-H$_2$O]$^-$ 945.5429[M-H-2CO$_2$-2Ac]$^-$ 927.5361[M-H-2CO2-2Ac-H$_2$O]$^-$ 765.4773[M-H-2CO$_2$-2Ac-H$_2$O-Glc]$^-$ 621.4378[M-H-2CO$_2$-2Ac-2Glc]$^-$ 459.3860[M-H-2CO$_2$-2Ac-3Glc]$^-$	PPD/PPD-C17-V4-Glc-Glc-Glc-malonyl-malonyl	D-malonyl-Rd	$C_{56}H_{86}O_{24}$	1118.5509	L5,L15,Y5,N5	1.34
27	6.2	869.4865	869.4844[M-H]$^-$ 825.5026[M-H-CO$_2$]$^-$ 783.4917[M-H-CO$_2$-Ac]$^-$ 793.4476 637.4411[M-H-CO$_2$-Ac-Rha]$^-$ 619.4101[M-H-CO$_2$-Ac-Rha-H$_2$O]$^-$ 475.3715[M-H-CO$_2$-Ac-Rha-Glc]$^-$	PPT-Glc-Rha-malonyl	malonyl-Rg2	$C_{45}H_{74}O_{16}$	870.4977	L5,L15,N5	3.91
28	6.39	797.4661	797.4844[M-H]$^-$ 635.4112[M-H-Glc]$^-$ 617.4116[M-H-Glc-H$_2$O]$^-$ 473.3679[M-H-2Glc]$^-$	PPT-C17-V5-Glc-Glc	Rh-2G1	$C_{42}H_{70}O_{14}$	798.4766	L5,L15,Y5,N5	3.39
29	6.68	959.523	959.5224[M-H]$^-$ 797.4642[M-H-Glc]$^-$ 635.4078[M-H-2Glc]$^-$ 473.3716[M-H-3Glc]$^-$	PPT-C17-V5-Glc-Glc-Glc	Rh-3G	$C_{48}H_{80}O_{19}$	960.5249	L5,L15	-6.15
30	7.29	1091.6066	1091.6066[M-H]$^-$ 946.5538[M-H-Rha]$^-$ 799.4800[M-H-2Rha]$^-$ 637.4425[M-H-2Rha-Glc]$^-$ 475.3918[M-H-2Rha-2Glc]$^-$	PPT-Glc-Glc-Rha-Rha	Re-G	$C_{54}H_{92}O_{22}$	1092.608	L5,L15	-5.86
31	7.44	987.5562	987.5562[M+FA-H]$^-$ 945.5393[M+FA-H-Ac]$^-$ 927.5407[M+FA-H-Ac-H2O]$^-$ 783.5059[M+FA-H-Ac-Glc]$^-$ 637.4343[M+FA-H-Ac-Glc-Rha]$^-$ 571.3929 459.3821[M-H-Ac-Glc-Rha-Xyl/Ara]$^-$	PPD/PPD-C17-V4-Xyl/Ara-Rha-Glc-acetyl	acetyl-Rh2-XR	$C_{49}H_{82}O_{17}$	942.5552	L5,L15,Y5,N5	-3.50
32	7.45	1019.5413	1019.5463[M+FA-H]$^-$ 973.5403[M-H]$^-$ 931.5293[M-H-Ac]$^-$ 799.4878[M-H-Ac-Xyl/Ara]$^-$ 637.4327[M-H-Ac-Xyl/Ara-Glc]$^-$ 475.3585[M-H-Ac-Xyl/Ara-2Glc]$^-$	PPT-Glc-Glc-Xyl/Ara-acetyl	acetyl-NG-R1B	$C_{49}H_{82}O_{19}$	974.545	L15	-3.18

33	7.8	843.4773	843.4719[M+FA-H]⁻ 797.4668[M-H]⁻ 635.4044[M-H-Glc]⁻ 473.3833[M-H-2Glc]⁻	PPT-C17-V5-Glc-Glc	Rh-2G2	$C_{42}H_{70}O_{14}$	798.4766	L5,L15,Y5,N5	2.51
34	7.84	913.515	913.5058[M-H]⁻ 781.4751[M-H-Xyl/Ara]⁻ 619.4176[M-H-Xyl/Ara-Glc]⁻ 457.3746[M-H-Xyl/Ara-2Glc]⁻	PPT-C17-V1/V2-Glc-Glc-Xyl/Ara	DHDXG-G2	$C_{47}H_{78}O_{17}$	914.5239	L15	1.20
35	7.9	1029.5249	1029.5187[M-H]⁻ 985.5372[M-H-CO_2]⁻ 943.5307[M-H-CO_2-Ac]⁻ 781.4715[M-H-CO_2-Ac-Glc]⁻ 619.4207[M-H-CO_2-Ac-2Glc]⁻ 457.3752[M-H-CO_2-Ac-3Glc]⁻	PPT-C17-V1/V2-Glc-Glc-Glc-malonyl	malonyl-DHD3G	$C_{51}H_{82}O_{21}$	1030.5349	L5,L15,Y5,N5	2.14
36	7.98	985.5349	985.5349[M-H]⁻ 943.5256[M-H-Ac]⁻ 935.5104 781.4708[M-H-Ac-Glc]⁻ 763.4356[M-H-Ac-Glc-H_2O]⁻ 619.4235[M-H-Ac-2Glc]⁻ 457.3626[M-H-Ac-3Glc]⁻	PPT-C17-V1/V2-Glc-Glc-Glc-acetyl	acetyl-DXD3G	$C_{50}H_{82}O_{19}$	986.545	L5,L15,Y5,N5	2.33
37	8.19	975.5574	929.5482[M-H]⁻ 783.4916[M-H-Rha]⁻ 765.4714[M-H-Rha-H_2O]⁻ 637.4445[M-H-2Rha]⁻ 619.4189[M-H-2Rha-H_2O]⁻ 475.3790[M-H-2Rha-Glc]⁻	PPD/PPD-C17-V4-Glc-Rha-Rha	C-K-2R	$C_{48}H_{82}O_{17}$	930.5552	L5,L15,Y5,N5	-0.86
38	8.29	1099.57	1099.5700[M-H]⁻ 1055.5461[M-H-CO_2]⁻ 987.5577[M-H-CO_2-C_4H_4O]⁻ 945.5549[M-H-CO_2-C_4H_4O-Ac]⁻ 783.4609[M-H-CO_2-Ac-C_4H_4O-Glc]⁻ 621.4250[M-H-CO_2-Ac-Glc-Glc]⁻ 459.4136[M-H-CO_2-Ac-C_4H_4O-3Glc]⁻	PPD/PPD-C17-V4-Glc-Glc-Glc-butene acyl-acetyl	butene acyl-acetyl-Rd1	$C_{55}H_{88}O_{22}$	1100.5767	L5,L15,Y5,N5	-1.00
39	8.38	973.5338	973.5386[M+FA-H]⁻ 927.5333[M-H]⁻ 781.4906[M-H-Rha]⁻ 619.4229[M-H-Rha-Glc]⁻ 457.3663[M-H-Rha-2Glc]⁻	PPT-C17-V1/V2-Glc-Glc-Rha	RG6-G2	$C_{48}H_{80}O_{17}$	928.5396	L5,L15,Y5,N5	-1.62
40	8.66	1055.5735	1055.5735[M-H]⁻ 987.5559[M-H-C_4H_4O]⁻ 945.5475[M-H-C_4H_4O-Ac]⁻ 783.4845[M-H-C_4H_4O-Ac-Glc]⁻ 621.4413[M-H-C_4H_4O-Ac-2Glc]⁻ 459.3570[M-H-C_4H_4O-Ac-3Glc]⁻	PPD/PPD-C17-V4-Glc-Glc-Glc-butene acyl-acetyl	butene acyl-acetyl-Rd2	$C_{54}H_{88}O_{20}$	1056.5869	L5,L15,Y5,N5	5.31

41	9.06	973.5344	973.5344[M+FA+H]$^-$ 927.5315[M-H]$^-$ 799.4842[M-H-Rha+H_2O]$^-$ 637.4384[M-H-Rha-Glc+H_2O]$^-$ 475.3809[M-Rha-2Glc+H_2O]$^-$ 457.3656[M-H-Rha-2Glc]$^-$	PPT-C17-V1/V2-Glc-Glc-Rha	RG6-G3	$C_{48}H_{80}O_{17}$	928.5396	L5,L15,Y5,N5	0.32
42	9.49	797.469	797.4732[M-H]$^-$ 635.4156[M-H-Glc]$^-$ 473.3723[M-H-2Glc]$^-$	PPT-C17-V5-Glc-Glc	Rh-2G3	$C_{42}H_{70}O_{14}$	798.4766	L5,L15,Y5,N5	-0.25
43	9.61	999.5537	999.5562[M-H]$^-$ 931.5240[M-H-C_4H_4O]$^-$ 799.5457[M-H-C_4H_4O-Xyl/Ara]$^-$ 637.4231[M-H-C_4H_4O-Xyl/Ara-Glc]$^-$ 475.3681[M-H-C_4H_4O-Xyl/Ara-2Glc]$^-$	PPT-Glc-Glc-Xyl/Ara-butene acyl	butene acyl-NG-R1	$C_{51}H_{84}O_{19}$	1000.5607	L15,Y5	-0.80
44	9.81	1089.5816	1089.5887[M-H_2O-H]$^-$ 945.5582[M-H-Glc]$^-$ 927.5169[M-H-Glc-H_2O]$^-$ 769.4679[M-H-Glc-GlcA]$^-$ 637.4359[M-H-Glc-GlcA-Ara/Xyl]$^-$ 475.3746[M-H-2Glc-GlcA-Ara/Xyl]$^-$	PPT-Glc-GlcA-Xyl/Ara-Glc	NG-R1-GA	$C_{53}H_{88}O_{24}$	1108.5666	L5,L15,N5	-1.08
45	9.85	883.4658	883.4760[M+FA-H]$^-$ 837.4935[M-H]$^-$ 769.4886[M-H-C_4H_4O]$^-$ 637.4508[M-H-C_4H_4O-Xyl/Ara]$^-$ 475.3743[M-H-C_4H_4O-Xyl/Ara-Glc]$^-$	PPT-Glc-Xyl/Ara-butene acyl	butene acyl-NG-R2	$C_{45}H_{74}O_{14}$	838.5079	L5,L15,Y5,N5	7.88
46	9.9	1091.5655	1091.5655[M-H]$^-$ 915.5721[M-H-GlcA]$^-$ 783.4803[M-H-GlcA-Xyl/Ara]$^-$ 621.4347[M-H-GlcA-Xyl/Ara-Glc]$^-$ 459.3908[M-H-GlcA-Xyl/Ara-2Glc]$^-$	PPD/PPD-C17-V4-Glc-Glc-Xyl/Ara-GlcA	CS-III-GA	$C_{53}H_{88}O_{23}$	1092.5716	L15,Y5,N5	-1.56
47	9.91	957.5433	957.5450[M-H]$^-$ 915.5336[M-H-Ac]$^-$ 765.4829[M-H-Ac-Ara/Xyl-H_2O]$^-$ 621.4384[M-H-Ac-Ara/Xyl-Glc]$^-$ 459.3877[M-H-Ac-Ara/Xyl-2Glc]$^-$	PPD/PPD-C17-V4-Glc-Glc-Xyl/Ara-acetyl	acetyl-CS-IIIA	$C_{49}H_{82}O_{18}$	958.5501	L5,L15,Y5,N5	-1.04
48	9.91	1001.5334	1001.5341[M-H]$^-$ 957.5440[M-H-CO_2]$^-$ 915.5331[M-H-CO_2-Ac]$^-$ 897.5227[M-H-CO_2-Ac-H_2O]$^-$ 783.4886[M-H-Ac-GlcA]$^-$ 765.4796[M-H-Ac-GlcA-H_2O]$^-$ 621.4360[M-H-Ac-GlcA-Glc]$^-$ 459.3908[M-H-Ac-GlcA-2Glc]$^-$	PPD/PPD-C17-V4-Glc-Glc-GlcA-acetyl	acetyl-F2-GA1	$C_{50}H_{82}O_{20}$	1002.5399	L5,L15,Y5,N5	-1.30

49	9.97	1105.5767	1105.5853[M-H]$^-$ 943.5257[M-H-Glc]$^-$ 781.4860[M-H-2Glc]$^-$ 619.4215[M-H-3Glc]$^-$ 457.3757[M-H-4Glc]$^-$	PPT-C17-V1/V2-Glc-Glc-Glc-Glc	DHD4G	$C_{54}H_{90}O_{23}$	1106.5873	L5,L15,Y5,N5	2.53
50	10.15	973.573	973.5730[M-H]$^-$ 945.5348[M-H-CO]$^-$ 783.4910[M-H-CO-Glc]$^-$ 637.4468[M-H-CO-Glc-Rha]$^-$ 475.4179[M-H-CO-Glc-Rha-Glc]$^-$	PPT-Glc-Glc-Rha-formyl	formyl-F2-R	$C_{50}H_{86}O_{18}$	974.5814	Y5,N5	0.62
51	10.17	767.4604	767.4543[M-H]$^-$ 635.4190[M-H-Xyl/Ara]$^-$ 473.3615[M-H-Xyl/Ara-Glc]$^-$	PPT-C17-V5-Glc-Xyl/Ara	Rh-GX1	$C_{41}H_{68}O_{13}$	768.466	Y5,N5	-2.87
52	10.22	957.5418	957.5405[M-H]$^-$ 915.5364[M-H-Ac]$^-$ 783.4952[M-H-Ac-Ara/Xyl]$^-$ 621.4454[M-H-Ac-Ara/Xyl-Glc]$^-$ 459.3717[M-H-Ac-Ara/Xyl-2Glc]$^-$	PPD/PPD-C17-V4-Glc-Glc-Xyl/Ara-acetyl	acetyl-CS-IIIB	$C_{49}H_{82}O_{18}$	958.5501	L5,L15,Y5,N5	0.52
53	10.31	1001.5343	1001.5397[M-H]$^-$ 957.5495[M-H-CO_2]$^-$ 915.5383[M-H-CO_2-Ac]$^-$ 783.4975[M-H-Ac-GlcA]$^-$ 765.4897[M-H-Ac-GlcA-H_2O]$^-$ 621.4340[M-H-Ac-GlcA-Glc]$^-$ 459.3897[M-H-Ac-GlcA-2Glc]$^-$	PPD/PPD-C17-V4-Glc-Glc-GlcA-acetyl	acetyl-F2-GA2	$C_{50}H_{82}O_{20}$	1002.5399	L5,L15,Y5,N5	-2.20
54	10.39	1013.5667	1013.5721[M-H]$^-$ 945.5460[M-H-C_4H_4O]$^-$ 927.5272[M-H-C_4H_4O-H_2O]$^-$ 799.4930[M-H-C_4H_4O-Rha]$^-$ 637.4264[M-H-C_4H_4O-Rha-Glc]$^-$ 475.3868[M-H-C_4H_4O-Rha-2Glc]$^-$	PPT-Glc-Glc-Rha-butene acyl	butene acyl-F2-R	$C_{52}H_{86}O_{19}$	1014.5763	L5,L15,Y5,N5	1.78
55	10.82	1073.5571	1073.5555[M-H]$^-$ 1029.5678[M-H-CO_2]$^-$ 987.5572[M-H-CO_2-Ac]$^-$ 969.5451[M-H-CO_2-Ac-H_2O]$^-$ 945.5452[M-H-CO_2-2Ac]$^-$ 927.5335[M-H-CO_2-2Ac-H2O]$^-$ 909.5230[M-H-CO_2-2Ac-2H_2O]$^-$ 765.4762[M-H-CO_2-2Ac-H_2O-Glc]$^-$ 621.4462[M-H-CO_2-2Ac-2Glc]$^-$ 459.3963[M-H-CO_2-2Ac-3Glc]$^-$	PPD-Glc-Glc-Glc-malonyl-acetyl	acetyl-Rd	$C_{53}H_{86}O_{22}$	1074.5611	L5,L15,Y5,N5	-3.54
56	10.98	861.459	861.4590[M-H]$^-$ 793.4165[M-H-C_4H_4O]$^-$ 617.3394[M-H-C_4H_4O-GlcA]$^-$ 455.3483[M-H-C_4H_4O-GlcA-Glc]$^-$	OA-Glc-GlcA-butene acyl	butene acyl-CS-Iva	$C_{46}H_{70}O_{15}$	862.4715	L5,L15,Y5,N5	5.46
57	11.16	971.5612	971.5612[M-H]$^-$ 929.5514[M-H-Ac]$^-$ 911.5376[M-H-Ac-H_2O]$^-$ 765.4803[M-H-Ac-H_2O-Rha]$^-$ 621.4465[M-H-Ac-Rha-Glc]$^-$ 459.3873[M-H-Ac-Rha-2Glc]$^-$	PPD/C17(D)-V4-Glc-Glc-Rha-acetyl	acetyl-F2-R1	$C_{50}H_{84}O_{18}$	972.5658	L5,L15,Y5,N5	-3.29

58	11.16	1015.5488	1015.5528[M-H]$^-$ 971.5609[M-H-CO_2]$^-$ 929.5409[M-H-CO_2-Ac]$^-$ 911.5392[M-H-CO_2-Ac-H_2O]$^-$ 783.4926[M-H-CO_2-Ac-Rha]$^-$ 765.4818[M-H-CO_2-Ac-Rha-H_2O]$^-$ 621.4449[M-H-CO_2-Ac-Rha-Glc]$^-$ 459.3858[M-H-CO_2-Ac-Rha-2Glc]$^-$	PPD/PPD-C17-V4-Glc-Glc-Rha-malonyl	malonyl-F2-R	$C_{51}H_{84}O_{20}$	1016.5556	L5,L15,Y5,N5	-0.98
59	11.36	813.4626	813.4670[M+FA-H]$^-$ 767.4590[M-H]$^-$ 635.4168[M-H-Xyl/Ara]$^-$ 473.3540[M-H-Xyl/Ara-Glc]$^-$	PPT-C17-V5-Glc-Xyl/Ara	Rh-GX2	$C_{41}H_{68}O_{13}$	768.466	L15,Y5	-1.04
60	11.42	1179.6138	1179.6188[M-H]$^-$ 1047.5720[M-H-Ara/Xyl]$^-$ 915.5444[M-H-2Ara/Xyl]$^-$ 753.4945[M-H-2Ara/Xyl-Glc]$^-$ 621.4445[M-H-3Ara/Xyl-Glc]$^-$ 459.3824[M-H-3Ara/Xyl-2Glc]$^-$	PPD/PPD-C17-V4-Glc-Glc-Ara/Xyl-Ara/Xyl-Ara/Xyl	CS-III-X1	$C_{57}H_{96}O_{25}$	1180.6241	L5,L15,Y5,N5	2.12
61	11.68	1055.579	1055.5840[M-H]$^-$ 987.5533[M-H-C_4H_4O]$^-$ 945.5510[M-H-C_4H_4O-Ac]$^-$ 765.4825[M-H-C_4H_4O-Ac-Glc-H_2O]$^-$ 621.4443[M-H-C_4H_4O-Ac-2Glc]$^-$ 459.3762[M-H-C_4H_4O-Ac-3Glc]$^-$	PPD/PPD-C17-V4-Glc-Glc-Glc-acetyl-butene acyl	butene acid-Rc	$C_{54}H_{88}O_{21}$	1056.5869	L5,L15,Y5,N5	0.09
62	11.82	1091.5974	1091.6068[M-H]$^-$ 945.5576[M-H-Rha]$^-$ 783.4961[M-H-Rha-Glc]$^-$ 621.4470[M-H-Rha-2Glc]$^-$ 459.4131[M-H-Rha-3Glc]$^-$	PPD/PPD-C17-V4-Glc-Glc-Glc-Rha	Rd-R	$C_{54}H_{92}O_{22}$	1092.608	L5,L15,Y5,N5	2.57
63	12	1179.6154	1179.6168[M-H]$^-$ 1047.5746[M-H-Ara/Xyl]$^-$ 915.5237[M-H-2Ara/Xyl]$^-$ 753.4839[M-H-2Ara/Xyl-Glc]$^-$ 621.4416[M-H-3Ara/Xyl-Glc]$^-$ 459.3888[M-H-3Ara/Xyl-2Glc]$^-$	PPD/PPD-C17-V4-Glc-Glc-Ara/Xyl-Ara/Xyl-Ara/Xyl	CS-III-X2	$C_{57}H_{96}O_{25}$	1180.6241	L5,L15,Y5,N5	0.76
64	12.03	943.5281	943.5291[M-H]$^-$ 781.4732[M-H-Glc]$^-$ 619.4165[M-H-2Glc]$^-$ 457.3768[M-H-3Glc]$^-$	PPT-C17-V1/V2-Glc-Glc-Glc	DHD3G	$C_{48}H_{80}O_{18}$	944.5345	L5,L15,Y5,N5	-1.48

65	12.18	1055.5786	1055.5786[M-H]$^-$ 1013.5735[M-H-Ac]$^-$ 945.5372[M-H-Ac-C_4H_4O]$^-$ 765.4871[M-H-Ac-C_4H_4O-Glc-H2O]$^-$ 621.4685[M-H-Ac-C_4H_4O-2Glc]$^-$ 459.3911[M-H-Ac-C_4H_4O-3Glc]$^-$	PPD/PPD-C17-V4-Glc-Glc-Glc-acetyl-butene acyl	acetyl-butene acyl-Rd	$C_{54}H_{88}O_{22}$	1056.5869	L5,L15,Y5,N5	0.47
66	12.2	1099.5695	1099.5695[M-H]$^-$ 1055.5874[M-H-CO_2]$^-$ 1013.5702[M-H-CO_2-Ac]$^-$ 945.5487[M-H-CO_2-Ac-C_4H_4O]$^-$ 783.52797[M-H-CO_2-Ac-C_4H_4O-Glc]$^-$ 621.4371[M-H-CO_2-Ac-C_4H_4O-2Glc]$^-$ 459.3907[M-H-CO_2-Ac-C_4H_4O-3Glc]$^-$	PPD/PPD-C17-V4-Glc-Glc-Glc-butene acyl-malonyl	butene acyl-malonyl-Rd	$C_{55}H_{88}O_{22}$	1100.5767	L5,L15,Y5,N5	-0.55
67	12.3	799.4819	799.4771[M+FA-H]$^-$ 753.4806[M-H]$^-$ 607.4226[M-H-Rha]$^-$ 475.3784[M-H-Rha-Xyl/Ara]$^-$	PPT-Xyl/Ara-Rha	PPTXR	$C_{41}H_{70}O_{12}$	754.4867	L15,N5	-2.26
68	13.84	837.4989	837.5072[M-H]$^-$ 769.4752[M-HC_4H_4O]$^-$ 637.4264[M-H-C_4H_4O-Xyl/Ara]$^-$ 475.3821[M-H-C_4H_4O-Xyl/Ara-Glc]$^-$	PPT-Glc-Xyl/Ara-butene acyl	butene acyl-F3	$C_{45}H_{74}O_{14}$	838.5079	L5,L15,Y5	1.43
69	13.89	851.4765	807.4902[M-H-CO_2]$^-$ 765.4788[M-H-CO_2-Ac]$^-$ 747.4723[M-H-CO_2-Ac-H_2O]$^-$ 603.4176[M-H-CO_2-Ac-Glc]$^-$ 441.3685[M-H-CO_2-Ac-2Glc]$^-$	PPD-C17-V1/V2-Glc-Glc-malonyl	malonyl-Rk1	$C_{45}H_{72}O_{15}$	852.4871	L5,L15LY5LN5	3.29
70	14.06	681.4182	681.4309[M+FA-H]$^-$ 635.4208[M-H]$^-$ 473.3624[M-H-Glc]$^-$	PPT-C17-V5-Glc	Rh6	$C_{36}H_{60}O_9$	636.4237	L5,L15,Y5	-7.71
71	14.91	1145.6067	1145.6066[M-H]$^-$ 1077.6177[M-H-C_4H_4O]$^-$ 945.5502[M-H-C_4H_4O-Xyl/Ara-]$^-$ 799.5008[M-H-C4H4O-Xyl/Ara-Rha]$^-$ 781.4819[M-H-C_4H_4O-Xyl/Ara-Rha-H_2O]$^-$ 637.4499[M-H-C_4H_4O-Xyl/Ara-Rha-Glc]$^-$ 475.3885[M-H-C_4H_4O-Xyl/Ara-Rha-2Glc]$^-$	PPT-Glc-Glc-Rha-Xyl/Ara-butene acyl	butene acyl-Re-X	$C_{57}H_{94}O_{23}$	1146.6186	L5,L15,Y5,N5	3.58
72	14.99	957.5413	957.5446[M-H]$^-$ 915.5333[M-H-Ac]$^-$ 783.4858[M-H-Ac-Xyl/Ara]$^-$ 621.4494[M-H-Ac-Xyl/Ara-Glc]$^-$ 459.3788[M-H-Ac-Xyl/Ara-2Glc]$^-$	PPD/PPD-C17-V4-Glc-Glc-Xyl/Ara-acetyl	acetyl-CS-IIIC	$C_{49}H_{82}O_{18}$	958.5501	L5,L15,Y5,N5	1.04
73	15.19	665.4269	665.4262[M+FA-H]$^-$ 619.4147[M-H]$^-$ 473.4978[M-H-Rha]$^-$	PPT-C17-V5-Rha	RH7	$C_{36}H_{60}O_8$	620.4288	L5,L15,Y5,N5	10.00

74	16.52	971.5564	971.5585[M-H]$^-$ 929.5492[M-H-Ac]$^-$ 911.5554[M-H-Ac-H_2O]$^-$ 783.4815[M-H-Ac-Rha]$^-$ 621.4260[M-H-Ac-Rha-Glc]$^-$ 459.2371[M-H-Ac-Rha-2Glc]$^-$	PPD/C17(D)-V4-Glc-Glc-Rha-acetyl	acetyl-F2-R2	$C_{50}H_{84}O_{18}$	972.5658	L5,L15,N5	1.65
75	25.68	667.4005	667.4005[M-H]$^-$ 491.3416[M-H-GlcA]$^-$	PG-GlcA	PG-GA	$C_{36}H_{60}O_{11}$	668.4136	L5,L15,Y5	7.94

Table S3 Semi-quantitative contents of 312 ginsenosides

Rt	L15	Y5	N5	L5	Rt	L15	Y5	N5	L5
1.18	4.90E+04	1.10E+05	8.50E+04	4.10E+04	9.06	6.00E+04	8.10E+04	4.80E+04	1.10E+05
1.25	2.60E+04	4.20E+04	8.20E+03	9.80E+03	9.06	3.90E+04	3.20E+04	3.30E+04	5.20E+04
1.37	1.60E+04	3.00E+04	2.20E+04	9.90E+03	9.08	3.60E+04	4.70E+04	2.50E+04	3.40E+04
1.39	1.10E+04	4.50E+04	1.80E+04	0.00E+00	9.09	7.10E+06	6.70E+06	4.50E+06	3.30E+06
1.40	1.00E+04	4.70E+04	1.60E+04	3.00E+03	9.09	4.30E+06	3.10E+06	2.60E+06	1.80E+06
1.48	0.00E+00	0.00E+00	0.00E+00	4.10E+03	9.09	4.30E+06	3.10E+06	2.60E+06	1.80E+06
1.50	4.90E+04	4.90E+04	4.10E+04	2.20E+04	9.15	1.60E+04	1.50E+04	1.20E+04	6.10E+03
1.54	0.00E+00	4.30E+03	0.00E+00	0.00E+00	9.49	3.60E+03	3.70E+03	2.10E+03	3.20E+03
1.62	6.60E+04	5.60E+04	4.30E+05	1.60E+04	9.52	1.50E+03	4.40E+03	1.80E+03	2.30E+03
1.62	4.70E+04	2.60E+04	3.40E+04	1.10E+04	9.61	0.00E+00	3.70E+03	1.60E+03	0.00E+00
1.63	1.40E+04	2.70E+05	3.80E+05	3.40E+04	9.66	3.50E+04	2.60E+03	9.30E+03	8.00E+03
1.69	1.40E+04	0.00E+00	1.20E+04	8.30E+03	9.67	0.00E+00	1.60E+04	3.10E+03	1.80E+03
1.79	7.20E+04	5.20E+04	3.30E+05	4.10E+04	9.75	3.40E+03	7.50E+03	4.90E+03	6.60E+03
1.87	4.50E+03	3.70E+03	2.70E+03	0.00E+00	9.77	0.00E+00	6.90E+04	5.60E+04	9.20E+04
1.89	1.80E+06	1.00E+06	8.40E+05	8.00E+04	9.78	2.70E+04	3.80E+04	2.20E+04	1.80E+04
1.90	2.70E+04	1.40E+04	2.80E+04	3.10E+04	9.81	1.20E+04	1.10E+04	0.00E+00	3.30E+03
1.90	2.70E+04	1.40E+04	2.80E+04	3.10E+04	9.83	1.00E+06	5.40E+06	2.50E+06	2.40E+05
1.93	7.70E+03	5.50E+04	0.00E+00	0.00E+00	9.83	2.80E+05	2.70E+06	1.40E+06	1.10E+05
1.95	2.90E+04	1.60E+04	1.50E+04	1.70E+04	9.85	1.70E+04	1.00E+04	1.70E+04	8.70E+04
1.96	2.70E+04	1.20E+04	2.50E+04	1.20E+04	9.90	0.00E+00	4.70E+04	2.20E+04	1.50E+04
2.02	1.40E+04	1.10E+05	6.90E+04	2.00E+04	9.91	3.30E+05	3.00E+05	2.40E+05	2.30E+05
2.21	9.90E+04	7.20E+04	4.80E+04	5.70E+05	9.91	1.50E+05	1.60E+05	1.30E+05	1.60E+05
2.21	1.10E+05	1.00E+06	3.60E+04	5.80E+05	9.97	6.40E+04	1.70E+05	5.80E+04	3.80E+04
2.25	9.00E+04	3.70E+05	6.00E+05	1.30E+06	10.15	0.00E+00	0.00E+00	3.10E+03	2.30E+03
2.34	5.10E+04	3.80E+04	2.00E+04	6.70E+03	10.17	0.00E+00	0.00E+00	1.80E+03	1.20E+03
2.36	0.00E+00	0.00E+00	0.00E+00	3.10E+03	10.22	3.60E+04	2.70E+04	2.10E+04	2.50E+04
2.48	3.40E+03	0.00E+00	2.50E+03	2.90E+03	10.25	1.10E+05	2.10E+05	9.60E+04	1.20E+05
2.72	1.50E+03	5.50E+03	3.50E+03	1.30E+03	10.31	2.10E+04	3.00E+04	2.40E+04	2.20E+04
2.74	1.30E+04	1.20E+04	6.20E+03	1.10E+04	10.39	8.50E+03	5.20E+03	1.10E+04	8.60E+03
2.77	1.10E+04	9.80E+03	5.10E+03	1.80E+04	10.39	5.90E+03	2.60E+03	6.60E+03	8.90E+03
2.82	4.70E+03	4.20E+03	1.80E+03	1.60E+03	10.42	0.00E+00	3.60E+04	3.30E+03	7.20E+04
2.83	1.80E+04	1.10E+04	1.30E+04	3.60E+03	10.42	1.40E+05	1.60E+05	6.10E+04	7.70E+04
2.87	3.70E+04	0.00E+00	2.20E+04	1.60E+04	10.47	6.00E+03	1.80E+04	8.90E+03	0.00E+00
3.00	7.10E+04	9.40E+04	2.00E+04	2.10E+04	10.49	7.70E+06	1.10E+07	6.20E+06	6.80E+06
3.20	1.80E+04	2.40E+04	8.40E+03	3.60E+03	10.55	1.30E+06	9.50E+05	7.90E+05	1.20E+06
3.30	0.00E+00	4.70E+03	0.00E+00	0.00E+00	10.55	1.30E+06	9.50E+05	7.90E+05	1.20E+06
3.35	0.00E+00	6.20E+03	4.30E+03	3.70E+03	10.64	2.90E+04	2.80E+04	1.90E+04	1.20E+04
3.36	0.00E+00	8.30E+04	2.70E+03	3.20E+03	10.64	4.60E+04	5.10E+04	2.90E+04	2.90E+04
3.38	1.30E+05	6.30E+04	1.60E+04	4.20E+04	10.78	0.00E+00	0.00E+00	0.00E+00	2.10E+03
3.67	7.80E+03	4.60E+04	2.20E+04	1.20E+04	10.82	9.40E+04	8.00E+04	5.20E+04	1.90E+05
3.68	3.60E+05	6.10E+05	3.10E+05	1.70E+05	10.84	0.00E+00	3.00E+03	0.00E+00	6.70E+03

3.82	0.00E+00	9.30E+03	5.30E+03	9.40E+02	10.97	1.00E+04	2.50E+04	1.50E+04	6.70E+03
3.97	0.00E+00	3.50E+04	5.90E+03	0.00E+00	10.97	2.30E+05	1.80E+05	1.60E+05	1.50E+05
4.15	0.00E+00	2.10E+03	0.00E+00	0.00E+00	10.98	4.50E+03	3.10E+03	2.50E+03	2.20E+03
4.48	1.60E+05	2.40E+06	9.60E+05	4.00E+05	11.04	1.30E+05	2.40E+05	7.90E+04	1.50E+05
4.61	1.80E+03	2.60E+05	9.70E+02	1.30E+05	11.06	1.80E+06	3.00E+06	1.20E+06	2.20E+06
4.64	1.70E+05	1.50E+05	8.70E+04	3.70E+04	11.12	4.50E+03	6.50E+03	3.30E+03	2.40E+03
4.66	4.60E+03	1.50E+03	4.90E+03	6.50E+03	11.12	4.50E+03	6.50E+03	3.30E+03	2.40E+03
4.68	0.00E+00	2.20E+04	7.40E+03	0.00E+00	11.16	8.90E+04	6.00E+04	4.10E+04	3.90E+04
4.68	2.30E+04	3.30E+03	4.90E+04	8.60E+03	11.16	4.50E+04	3.60E+04	1.70E+04	2.60E+04
4.73	2.30E+06	1.80E+06	6.10E+05	3.00E+05	11.19	2.90E+03	3.70E+03	2.20E+03	2.10E+03
4.81	0.00E+00	0.00E+00	3.90E+03	1.60E+03	11.36	0.00E+00	5.30E+03	4.00E+03	0.00E+00
4.82	4.30E+04	4.00E+04	3.40E+04	2.40E+04	11.42	3.20E+04	5.00E+04	2.20E+04	1.40E+04
4.85	7.20E+03	2.40E+05	6.00E+04	0.00E+00	11.43	9.00E+03	5.60E+03	5.90E+03	2.20E+03
4.90	7.50E+03	9.00E+03	3.50E+03	4.90E+03	11.55	5.70E+06	5.50E+06	3.90E+06	4.10E+06
4.91	5.40E+03	9.70E+03	3.90E+03	4.40E+03	11.55	5.70E+06	5.50E+06	3.90E+06	4.10E+06
4.91	5.00E+03	5.70E+03	2.90E+03	3.90E+03	11.58	6.30E+03	4.30E+03	4.50E+03	2.80E+04
4.94	5.30E+04	3.00E+04	1.40E+04	1.00E+04	11.60	3.10E+03	3.20E+03	2.50E+03	0.00E+00
5.04	3.60E+03	3.00E+03	1.40E+03	4.00E+03	11.68	1.60E+04	8.70E+03	1.50E+04	1.40E+04
5.19	8.00E+04	0.00E+00	0.00E+00	0.00E+00	11.77	6.50E+04	2.40E+04	1.40E+04	2.30E+04
5.19	3.30E+05	3.40E+06	1.20E+06	6.60E+04	11.82	4.30E+04	3.70E+04	1.40E+04	2.10E+04
5.22	8.80E+04	8.20E+04	7.80E+04	8.10E+04	11.82	3.30E+04	3.30E+04	1.80E+04	2.20E+04
5.23	1.20E+04	6.80E+03	0.00E+00	0.00E+00	11.91	3.00E+03	3.60E+03	2.00E+04	5.90E+03
5.24	0.00E+00	2.50E+04	1.50E+04	5.00E+03	11.97	7.10E+03	2.90E+03	0.00E+00	6.90E+03
5.27	0.00E+00	5.00E+03	2.10E+03	0.00E+00	12.00	4.00E+04	1.00E+05	4.00E+04	3.00E+04
5.28	0.00E+00	0.00E+00	0.00E+00	6.60E+03	12.00	0.00E+00	1.10E+04	7.60E+03	0.00E+00
5.30	4.80E+04	4.50E+04	0.00E+00	3.70E+04	12.03	1.60E+04	2.50E+04	1.70E+04	3.30E+04
5.34	3.60E+05	4.60E+05	4.80E+05	2.10E+05	12.07	2.90E+06	3.40E+06	9.50E+05	1.40E+06
5.35	1.40E+04	9.90E+03	1.10E+04	9.40E+03	12.08	3.80E+04	3.20E+04	1.30E+04	5.10E+04
5.44	4.70E+05	9.90E+04	1.80E+05	1.40E+05	12.10	1.70E+04	3.30E+04	1.50E+04	1.40E+04
5.46	2.10E+04	2.90E+04	1.50E+04	1.90E+04	12.14	1.50E+04	6.30E+04	3.50E+04	1.90E+04
5.47	1.30E+06	3.50E+05	4.10E+05	3.80E+05	12.17	7.20E+03	0.00E+00	5.60E+03	2.20E+03
5.52	2.00E+04	1.10E+05	7.60E+04	1.60E+04	12.18	2.20E+04	9.70E+03	1.40E+04	8.20E+03
5.54	8.40E+03	5.50E+04	2.20E+04	6.60E+04	12.20	2.30E+04	1.30E+04	2.70E+04	2.20E+04
5.60	1.70E+03	1.70E+04	0.00E+00	5.30E+04	12.30	7.00E+03	1.90E+03	3.40E+03	2.60E+03
5.62	0.00E+00	6.50E+03	0.00E+00	0.00E+00	12.30	0.00E+00	2.20E+03	0.00E+00	2.00E+03
5.76	1.20E+04	1.20E+04	0.00E+00	0.00E+00	12.31	1.60E+04	1.30E+04	4.60E+04	5.60E+03
5.76	1.20E+04	1.40E+04	0.00E+00	0.00E+00	12.36	1.00E+04	1.10E+04	1.60E+04	1.50E+04
5.77	1.30E+07	1.30E+07	1.00E+07	6.60E+06	12.40	3.30E+04	5.90E+03	2.10E+04	3.20E+04
5.81	5.40E+06	6.00E+06	1.30E+07	1.10E+07	12.45	1.10E+05	6.30E+05	3.20E+05	4.90E+05
5.92	5.60E+04	2.70E+05	1.80E+05	1.30E+04	12.56	2.90E+06	3.60E+06	2.40E+06	3.10E+06
5.94	0.00E+00	4.30E+04	1.60E+04	0.00E+00	12.68	0.00E+00	1.10E+04	3.10E+03	9.60E+03
6.03	7.10E+04	5.10E+04	9.00E+03	1.90E+04	12.76	1.80E+03	4.60E+03	1.80E+03	4.70E+03
6.07	1.90E+04	1.10E+04	0.00E+00	0.00E+00	12.79	6.70E+04	9.50E+04	4.40E+04	3.60E+04
6.09	7.40E+03	0.00E+00	5.30E+03	6.50E+03	12.90	7.10E+05	6.80E+05	5.50E+05	6.10E+05

6.09	0.00E+00	1.00E+04	5.70E+03	0.00E+00	12.90	7.10E+05	6.80E+05	5.50E+05	6.10E+05
6.12	2.70E+04	3.60E+04	9.80E+03	0.00E+00	12.99	6.60E+03	3.80E+03	3.20E+03	5.10E+03
6.13	4.90E+04	3.60E+04	3.60E+04	7.00E+04	13.00	2.60E+04	1.50E+04	1.20E+04	4.10E+04
6.14	0.00E+00	1.40E+05	7.10E+04	4.90E+05	13.05	5.10E+05	4.40E+05	3.70E+05	1.90E+05
6.14	0.00E+00	7.20E+04	9.10E+04	0.00E+00	13.18	6.20E+04	6.40E+04	3.80E+04	6.10E+04
6.15	6.40E+03	1.40E+06	1.90E+06	2.50E+06	13.19	3.90E+04	8.30E+03	1.60E+04	1.00E+04
6.15	0.00E+00	3.80E+04	0.00E+00	0.00E+00	13.42	9.50E+04	5.70E+04	1.50E+05	6.40E+04
6.16	7.30E+04	2.30E+05	2.60E+05	4.30E+05	13.45	7.10E+03	5.90E+04	2.40E+04	2.80E+04
6.18	5.70E+03	4.80E+04	1.60E+04	2.50E+04	13.49	1.80E+03	4.30E+03	3.70E+04	1.80E+04
6.19	6.10E+04	4.20E+04	0.00E+00	6.10E+04	13.57	9.90E+03	5.20E+03	2.70E+03	2.40E+03
6.20	8.90E+03	8.50E+03	8.10E+03	1.70E+04	13.77	1.30E+04	9.30E+03	2.80E+03	3.00E+03
6.20	1.80E+04	1.20E+04	9.50E+03	0.00E+00	13.78	1.10E+05	5.80E+05	1.90E+05	9.00E+05
6.26	0.00E+00	0.00E+00	1.20E+05	0.00E+00	13.78	7.10E+04	1.00E+05	4.30E+05	4.20E+04
6.29	0.00E+00	3.40E+04	4.30E+04	3.00E+04	13.81	3.90E+03	0.00E+00	0.00E+00	1.30E+03
6.29	1.20E+07	1.20E+07	1.10E+07	9.20E+06	13.84	1.90E+03	4.60E+03	5.10E+03	0.00E+00
6.37	0.00E+00	1.30E+04	0.00E+00	1.20E+04	13.89	1.10E+04	1.80E+04	9.10E+03	6.20E+03
6.39	5.60E+03	5.10E+03	1.10E+04	1.10E+04	13.89	1.20E+03	1.50E+03	2.10E+03	1.60E+03
6.43	8.30E+03	2.80E+03	0.00E+00	0.00E+00	14.06	2.00E+03	2.50E+03	2.50E+03	0.00E+00
6.51	6.20E+03	2.10E+04	1.20E+04	0.00E+00	14.08	4.90E+04	5.60E+04	1.90E+04	2.80E+04
6.52	1.10E+04	3.10E+04	3.50E+04	8.00E+03	14.10	1.10E+03	1.50E+03	0.00E+00	0.00E+00
6.54	0.00E+00	3.90E+03	3.80E+03	3.00E+03	14.26	4.20E+04	5.80E+04	3.00E+04	1.50E+05
6.54	0.00E+00	5.10E+03	3.40E+03	0.00E+00	14.39	3.50E+04	1.00E+04	6.80E+03	1.00E+04
6.64	0.00E+00	2.50E+04	0.00E+00	0.00E+00	14.39	1.40E+04	1.00E+04	2.80E+03	6.10E+03
6.68	6.70E+03	6.70E+03	0.00E+00	0.00E+00	14.46	2.30E+03	1.80E+04	3.80E+03	2.80E+03
6.74	1.70E+05	1.50E+05	7.30E+04	5.10E+04	14.50	9.30E+03	1.40E+04	5.10E+03	0.00E+00
6.81	1.20E+05	1.50E+05	1.40E+05	1.10E+05	14.50	1.80E+03	3.00E+03	1.20E+03	1.40E+03
6.95	0.00E+00	0.00E+00	5.90E+03	5.00E+03	14.68	6.40E+04	5.50E+04	7.30E+04	6.90E+04
6.98	5.00E+06	4.20E+06	5.00E+06	4.20E+06	14.68	3.80E+04	5.30E+04	2.00E+04	1.70E+04
7.04	1.00E+04	1.10E+04	5.40E+03	2.30E+04	14.68	4.20E+04	2.50E+04	1.60E+04	2.40E+04
7.08	5.20E+06	4.20E+06	3.60E+06	4.20E+06	14.83	8.80E+03	7.60E+03	2.90E+03	3.90E+03
7.13	2.70E+05	1.70E+05	1.10E+05	8.90E+04	14.83	8.80E+03	7.60E+03	2.90E+03	3.90E+03
7.13	2.50E+05	3.80E+05	4.00E+05	5.30E+05	14.91	1.70E+03	2.10E+03	3.40E+03	2.00E+03
7.17	7.60E+03	1.30E+04	2.00E+04	8.40E+03	14.98	2.70E+03	4.40E+03	2.80E+03	3.80E+03
7.27	0.00E+00	5.60E+04	1.80E+04	1.40E+04	14.99	1.40E+04	1.10E+04	2.90E+03	1.00E+04
7.29	2.50E+04	1.40E+04	0.00E+00	0.00E+00	15.19	1.10E+04	8.50E+03	4.20E+03	4.50E+03
7.36	2.10E+04	3.20E+04	1.50E+04	1.10E+04	15.34	1.80E+03	1.80E+03	1.00E+03	7.70E+02
7.41	1.20E+06	2.50E+06	2.20E+06	4.00E+06	15.39	1.10E+04	2.00E+04	1.90E+04	1.60E+04
7.44	1.90E+04	1.30E+04	6.00E+03	3.50E+03	15.60	8.40E+03	8.40E+03	2.60E+03	1.30E+04
7.45	0.00E+00	1.20E+04	0.00E+00	0.00E+00	16.16	0.00E+00	3.30E+03	9.90E+02	0.00E+00
7.48	7.90E+04	5.00E+04	8.80E+05	1.10E+06	16.18	2.90E+04	4.50E+04	9.70E+02	4.20E+04
7.49	0.00E+00	5.00E+04	3.90E+04	3.50E+04	16.18	2.90E+04	4.50E+04	9.70E+02	4.20E+04
7.52	6.40E+04	9.00E+04	0.00E+00	3.20E+04	16.46	5.90E+03	9.50E+03	3.80E+03	6.40E+03
7.54	1.50E+05	1.30E+05	1.10E+05	8.90E+04	16.52	1.10E+03	2.90E+03	0.00E+00	1.70E+03
7.65	0.00E+00	9.50E+03	5.70E+03	1.10E+03	16.97	1.70E+04	3.40E+04	1.70E+04	2.10E+04

7.69	5.50E+03	3.70E+03	3.00E+03	1.50E+03	17.01	1.10E+03	2.50E+03	1.30E+03	1.70E+03
7.71	2.50E+04	7.50E+04	4.20E+04	3.50E+04	17.01	1.10E+03	2.50E+03	1.30E+03	1.70E+03
7.76	0.00E+00	4.90E+03	1.70E+03	1.50E+03	17.18	5.30E+03	1.10E+04	1.80E+03	8.90E+03
7.80	1.40E+04	2.60E+04	1.30E+04	2.00E+04	17.35	2.40E+03	4.20E+03	2.20E+03	2.50E+03
7.80	1.20E+04	1.80E+04	9.70E+03	7.00E+03	17.55	5.80E+03	0.00E+00	7.70E+03	1.30E+04
7.84	0.00E+00	1.10E+04	0.00E+00	0.00E+00	17.70	1.30E+03	0.00E+00	1.90E+03	6.00E+03
7.90	2.90E+04	2.00E+04	2.20E+04	2.60E+04	18.33	2.10E+04	6.40E+03	3.30E+03	9.10E+03
7.97	9.50E+05	3.40E+05	7.10E+05	2.30E+05	18.33	2.10E+05	6.40E+03	3.30E+03	9.10E+03
7.97	0.00E+00	1.50E+04	2.00E+04	1.70E+04	18.44	0.00E+00	0.00E+00	0.00E+00	0.00E+00
7.98	1.80E+04	1.50E+04	1.50E+04	1.70E+04	18.44	0.00E+00	0.00E+00	0.00E+00	0.00E+00
8.09	6.40E+04	2.30E+06	2.60E+06	1.50E+06	18.45	1.60E+03	9.20E+02	2.00E+03	2.20E+03
8.19	6.40E+03	2.10E+04	1.60E+04	1.40E+04	19.06	7.10E+03	4.60E+03	1.10E+03	4.30E+03
8.19	1.60E+03	3.40E+03	2.20E+03	4.50E+03	19.07	0.00E+00	0.00E+00	0.00E+00	1.60E+03
8.25	9.20E+03	4.80E+03	3.80E+03	3.30E+03	19.90	3.40E+03	6.50E+02	9.30E+02	4.20E+03
8.29	3.00E+04	0.00E+00	1.80E+04	1.90E+04	19.93	3.60E+04	2.20E+04	5.60E+03	1.40E+04
8.31	5.90E+04	4.20E+04	4.40E+04	5.50E+04	20.06	3.40E+03	4.60E+03	6.30E+03	0.00E+00
8.34	1.70E+04	1.40E+04	7.70E+03	3.80E+03	20.68	1.40E+05	7.90E+04	1.50E+05	1.90E+04
8.38	1.60E+04	2.40E+04	2.10E+04	2.90E+04	21.02	1.40E+03	1.30E+03	1.40E+03	1.50E+03
8.40	1.60E+06	1.10E+06	9.20E+05	1.50E+06	21.02	1.40E+03	1.30E+03	1.40E+03	1.50E+03
8.60	1.10E+06	1.10E+06	3.20E+06	1.60E+06	21.29	1.80E+03	7.40E+02	2.90E+03	1.50E+03
8.66	4.10E+04	4.70E+04	2.50E+04	3.40E+04	21.68	2.90E+03	6.60E+03	4.00E+03	3.60E+03
8.69	0.00E+00	0.00E+00	0.00E+00	6.30E+03	21.91	4.40E+04	2.10E+04	1.10E+03	0.00E+00
8.75	3.50E+04	3.20E+04	2.00E+04	2.80E+04	23.72	1.20E+03	1.20E+03	1.30E+03	1.30E+03
8.77	1.50E+03	3.10E+04	1.30E+04	3.10E+03	23.72	1.20E+03	1.20E+03	1.30E+03	1.30E+03
8.90	5.40E+03	5.20E+03	1.70E+03	3.10E+03	25.54	3.70E+02	9.20E+02	8.80E+02	6.60E+02
8.98	9.20E+04	8.20E+04	7.90E+04	3.10E+04	25.54	3.70E+02	9.20E+02	8.80E+02	6.60E+02
9.06	7.20E+04	9.90E+04	8.80E+04	1.50E+05	25.68	1.10E+06	6.40E+05	2.40E+05	0.00E+00

Table S4 Ginseng samples analyzed in this study

Code no	Location	Time or Batch No.	Age (years)	category
L15JC1-24	Caiyuan Town Ji 'an, Jilin Province	PL15JC0124	15	Ginseng cultivated in the forest
L15JC2-2	Caiyuan Town Ji 'an, Jilin Province	PL15JI0202	15	Ginseng cultivated in the forest
L5JC1-1	Caiyuan Town Ji 'an, Jilin Province	PL05JC0101	5	Ginseng cultivated in the forest
L5JC2-4	Caiyuan Town Ji 'an, Jilin Province	PL05JC0204	5	Ginseng cultivated in the forest
L5JC4-1	Caiyuan Town Ji 'an, Jilin Province	PL05JC0401	5	Ginseng cultivated in the forest
Y5JC1-10	Caiyuan Town Ji 'an, Jilin Province	PY05JC0110	5	Ginseng cultivated in the mountains
Y5JT2-1	Taishang Town Ji 'an, Jilin Province	PY05JT0201	5	Ginseng cultivated in the mountains
Y5JC3-9	Caiyuan Town Ji 'an, Jilin Province	PY05JC0309	5	Ginseng cultivated in the mountains
N5JM1-1	Maxian Town Ji 'an, Jilin Province	PN05JM0101	5	Ginseng cultivated in farmland
N5JY2-2	Yulin Town Ji 'an, Jilin Province	PN05JY0202	5	Ginseng cultivated in farmland
N5JL3-2	Liangshui Town Ji 'an, Jilin Province	PN05JL0302	5	Ginseng cultivated in farmland

Table S5 Structure of the 63 ginsenoside standards

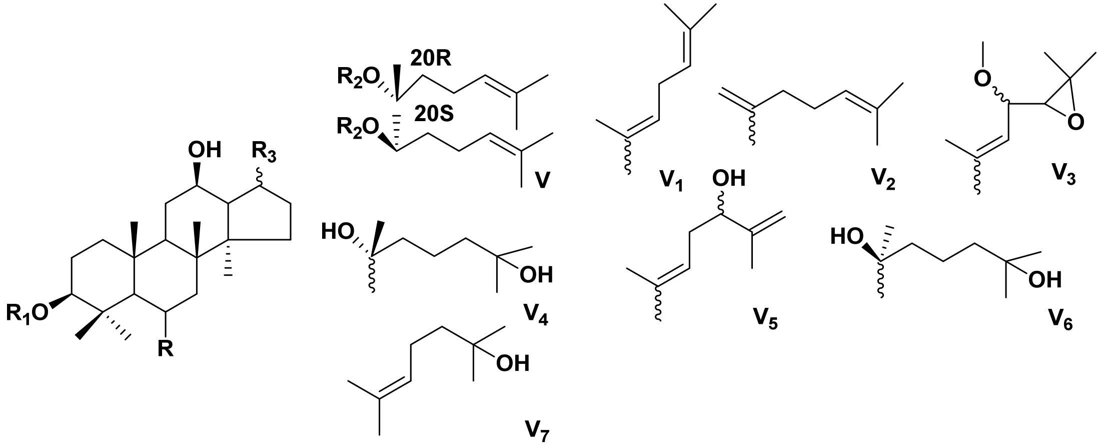

Protopanaxadiol&Protopanaxatriol (PPD&PPT)

No.	Compound	R	R1	R2	R3	No.	Compound	R	R1	R2	R3	No.	Compound	R	R1	R2	R3
12	NG R_4	-	-Glc(2-1)Glc	-Glc(6-1)Glc(6-1)Xyl	-V	26	20(S)-Rh_1	-O-Glc	-H	-H	-V	38	G Rg_6	-OGlc(2-1)Rha	-H	-	-V_2
18	G Ra_2	-	-Glc(2-1)Glc	-Glc(6-1)Ara(f)(2-1)Xyl	-V	27	20(R)-Rh_1	-O-Glc	-H	-H	-V	39	F_4	-OGlc(2-1)Rha	-H	-	-V_1

19	G Ra$_3$	-	-Glc(2-1)Glc	-Glc(6-1)Glc(3-1) Xyl	-V	7	G Rg$_1$	-O-Glc	-H	-Glc	-V	42	G Rk$_3$	-OGlc	-H	-	-V2
21	G Rb$_1$	-	-Glc(2-1)Glc	-Glc(6-1)Glc	-V	13	20(S)-Rf-1a	-OGlc(4-1)Glc	-H	-H	-V	43	G Rh$_4$	-Glc	-H	-	-V1
24	G Ra$_1$	-	-Glc(2-1)Glc	-Glc(6-1)Ara(p)(4-1) Xyl	-V	14	20(S)-Rf	-OGlc(2-1)Glc	-H	-H	-V	40	DHDXG	-OGlc(2-1)Xyl	-H	-	-V1
25	G Rc	-	-Glc(2-1)Glc	-Glc(6-1)Ara(f)	-V	15	20(R)-Rf	-OGlc(2-1)Glc	-H	-H	-V	44	G Rg$_9$	-OGlc(2-1)Glc	-H	-	-V1
28	G Rb$_2$	-	-Glc(2-1)Glc	-Glc(6-1)Ara(p)	-V	22	20(S)-Rg$_2$	-OGlc(2-1)Rha	-H	-H	-V	59	DDT	-OH	-H	-	-V2
29	G Rb$_3$	-	-Glc(2-1)Glc	-Glc(6-1Xyl)	-V	23	20(R)-Rg$_2$	-OGlc(2-1)Rha	-H	-H	-V	48	DEDT	-OH	-H	-	-V1
30	Q-R$_1$	-	-Glc(2-1)Glc(6)Ac	-Glc(6-1)Glc	-V	4	G Re$_2$	-OGlc(3-1)Glc	-H	-Glc	-V	53	Rs$_4$	-	-Glc(2-1)Glc(6)Ac	-	-V1
31	G Rd	-	-Glc(2-1)Glc	-Glc	-V	8	G Re	-OGlc(2-1)Rha	-H	-Glc	-V	32	Rk$_1$	-	-Glc(2-1)Glc	-	-V2

33	G Rs_2	-	-Glc(2-1)Glc(6)Ac	-Glc(6-1)Ara(f)	-V	2	20-glu-Rf	-OGlc(2-1)Glc	-H	-Glc	-V	55	Rg_5	-	-Glc(2-1)Glc	-	-V1
34	G Rs_1	-	-Glc(2-1)Glc(6)Ac	-Glc(6-1)Ara(p)	-V	6	G Re_1	-O-Glc	-H	-Glc(3-1)Glc	-V	37	DHDGG	-	-Glc(2-1)Glc	-	V7
45	G F_2	-	-Glc	-Glc	-V	1	G Re_3	-O-Glc	-H	-Glc(4-1)Glc	-V	60	Rh_3	-	-Glc	-	-V1
46	20-(S)-Rg_3	-	-Glc(2-1)Glc	-H	-V	3	G Re_4	-O-Glc	-H	-Glc(6-1)Ara(f)	-V	61	Rk_2	-	-Glc	-	-V2
47	20-(R)-Rg_3	-	-Glc(2-1)Glc	-H	-V	49	20(S)-PPT	-OH	-H	-H	-V						
51	20-(S)-Rs_3	-	-Glc(2-1)Glc(6)Ac	-H	-V	50	20(R)-PPT	-OH	-H	-H	-V						
52	20-(R)-Rs_3	-	-Glc(2-1)Glc(6)Ac	-H	-V	17	G F_3	-OH	-H	-Glc(6-1)Ara(p)	-V						
56	G-C-K	-	-H	-Glc	-V	5	NG R_1	-OGlc(2-1)Xyl	-H	-Glc	-V						
57	20-(S)-Rh_2	-	-Glc	-H	-V	16	20-(S)-NG-R_2	-OGlc(2-1)Xyl	-H	-H	-V						
58	20-(R)-Rh_2	-	-Glc	-H	-V	20	20-(R)-NG-R_2	-OGlc(2-1)Xyl	-H	-H	-V						
62	20-(S)-PPD	-	-H	-H	-V	36	G RH_{19}	-OH	-Glc	-H	-V						

63	20-(R)-PPD	-	-H	-H	-V

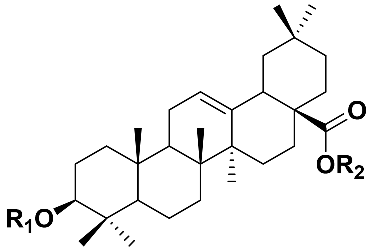

Oleanolic (OA)

NO.	Compound	R_1	R_2
35	G Ro methyl ester	-GlcA(6'-OMe,2-1)Glc	-Glc
9	G Ro	-GlcA(2-1)Glc	-Glc
41	CS-Iva methyl ester	-H	-GlcA(6'-OMe,2-1)Glc
54	ZB-RI-6‘ methyl ester	-GlcA(6'-OMe,2-1)Glc	-H
10	CS-IV	-GlcA(4-1)Ara(f)	-Glc
11	CS-Iva	-GlcA	-Glc
